# Heterotrimeric G-protein α subunit (*RGA1*) regulates tiller development, yield, cell wall, nitrogen response and biotic stress in rice

**DOI:** 10.1038/s41598-021-81824-1

**Published:** 2021-01-27

**Authors:** Ravi Ramesh Pathak, Vikas Kumar Mandal, Annie Prasanna Jangam, Narendra Sharma, Bhumika Madan, Dinesh Kumar Jaiswal, Nandula Raghuram

**Affiliations:** University School of Biotechnology, Guru Gobind Singh Indraprastha University, Sector 16C, Dwarka, New Delhi 110078 India

**Keywords:** Plant molecular biology, Plant signalling

## Abstract

G-proteins are implicated in plant productivity, but their genome-wide roles in regulating agronomically important traits remain uncharacterized. Transcriptomic analyses of rice G-protein alpha subunit mutant (*rga1*) revealed 2270 differentially expressed genes (DEGs) including those involved in C/N and lipid metabolism, cell wall, hormones and stress. Many DEGs were associated with root, leaf, culm, inflorescence, panicle, grain yield and heading date. The mutant performed better in total weight of filled grains, ratio of filled to unfilled grains and tillers per plant. Protein–protein interaction (PPI) network analysis using experimentally validated interactors revealed many *RGA1*-responsive genes involved in tiller development. qPCR validated the differential expression of genes involved in strigolactone-mediated tiller formation and grain development. Further, the mutant growth and biomass were unaffected by submergence indicating its role in submergence response. Transcription factor network analysis revealed the importance of *RGA1* in nitrogen signaling with DEGs such as Nin-like, WRKY, NAC, bHLH families, nitrite reductase, glutamine synthetase, OsCIPK23 and urea transporter. Sub-clustering of DEGs-associated PPI network revealed that RGA1 regulates metabolism, stress and gene regulation among others. Predicted rice G-protein networks mapped DEGs and revealed potential effectors. Thus, this study expands the roles of *RGA1* to agronomically important traits and reveals their underlying processes.

## Introduction

Heterotrimeric G proteins transduce external signals into intracellular responses through mechanisms that are well characterized in animals, but relatively less understood in plants^1,2^. In Arabidopsis, the Gα function may be regulated through the classical G-protein coupled receptor(s)^3–6^ or self-activation^7,8^ through the regulator of G-protein signaling (RGS) 1^9^ or through receptor-like kinases^1^. However, RGS proteins, though present in many grasses, there were frequent losses in different species like rice^10^. Genome annotation projects revealed the conservation of different subunits of heterotrimeric G-protein complexes in crop plants, including rice. Those functionally characterized so far in rice are, *RGA1* for Gα subunit^11–13^, *RGB1* for Gβ subunit^14,15^ and *RGG1*, *RGG2*^12,16^, *DEP1*, *GS3*, *GGC2* for Gγ subunits, respectively.

Functionally, some of the G-protein responses are predominantly mediated by the Gα subunit, whereas others are predominantly mediated by the Gβ and Gγ subunits. The roles attributed to the Gα subunit span morphological, developmental and physiological levels studied to various extents in different plants^17,18^. At the morphological level, Gα subunit has been implicated in the regulation of hypocotyl elongation, hook angle, rosette diameter, and leaf shape in *Arabidopsis*^19^. At the developmental level, Gα-regulation has been reported in pollen tube development^20^. At the physiological level, the processes regulated by Gα include ABA mediated stomatal opening^21^, stomatal density regulation^22^, sphingolipid signaling^23^, sugar signaling^24^, light-stimulation of nitrate reductase gene expression^25^ and phenylalanine and ABA production^26,27^. In rice, the reported roles of Gα include abiotic stresses^28^ including salt stress^29^, drought^30^, photo-oxidation^31^, plant immunity^32^, plant growth^33^, grain size^34^, nitrate response^35^ and brassinosteroid signaling^13,36^.

Functional genomic analyses of G-protein signaling mutants have been very useful in exploring their genome-wide roles in plants^5,6,28,31,37–40^. Microarray analysis of the Arabidopsis Gα mutant (*gpa1*) has revealed its major roles in stress, metabolism and nutrient response to nitrogen (N) and phosphorous, among others^6^. However, there are no comprehensive studies on the genome-wide role of G-proteins in any crop plant, despite their suggested roles in agronomically important traits. The availability of a natural mutant of the Gα subunit in rice^41^ provided such an opportunity for a long time and yet, the only large scale studies that attempted on it have been limited to photoprotection and photoavoidance^31^ and abiotic stresses^28,29^. The present study was aimed at a comprehensive genomewide analysis of the *rga1* mutant in comparison to its corresponding wild type to reveal many hitherto unknown roles of *rga1*, including regulation of many agronomically important traits and to identify their underlying processes.

## Results

### Transcriptome profiling and identification of differentially expressed genes

In this study, we have used the d1 (*rga1*) mutant, which was identified earlier^41^. We confirmed the down-regulation of *RGA1* transcript in the *rga1* mutant (Fig. S1). Microarray data analysis under MIAME compliant conditions using a fold change cut-off value of ± 1.0 (geometric mean log_2_) along with p-value cut off of ≤ 0.05 revealed a total of 2270 differentially expressed genes (DEGs) in the *rga1* mutant relative to WT^28^, with 1242 up-regulated and 1028 down-regulated genes, respectively (Fig. S2). Out of these, we earlier reported our analysis of a subset of these DEGs associated with abiotic stresses^28^; here we report a more comprehensive and in-depth analyses of all the identified DEGs with the latest available annotation information to understand the genomewide effect of *RGA1,* including on agronomically important traits and other pathways. The raw data are available at NCBI GEO (GSE20925). A plot of the number of DEGs against fold change (log_2_FC) revealed that the maximum number of DEGs fall within 1-2 fold change and significantly decrease with increasing fold change value (Fig. S2). A few DEGs showed 7-8 fold down-regulation or 5-6 fold up-regulation (Fig. S2).

### Gene ontology (GO) and MapMan pathway analyses of *RGA1*-responsive genes

To investigate the processes affected by the functional inactivation of *RGA1* in rice, the RAPDB database gene name of the DEGs were converted to RGAP database gene name using OryzaExpress ID converter tool (http://bioinf.mind.meiji.ac.jp/OryzaExpress/ID_converter.php). DEGs were subjected to functional annotation using singular enrichment analysis in the AgriGO database, which revealed the GO terms and associated FDR and *p*-values. The statistically enriched GO terms were used to visualize the different processes using TreeMap software. Among 2270 DEGs, 1781 could be broadly categorized into biological processes and cellular components (Fig. S2). The most enriched terms for biological processes were “response to stimulus”, “response to abiotic stimulus”, “response to biotic stimulus” and “metabolic process” among others. Cellular components analyses showed that the products of these DEGs were mainly distributed in the cell wall, extracellular region, membrane and various cellular organelles such as chloroplast, ribosomes etc.

**Figure 1:**
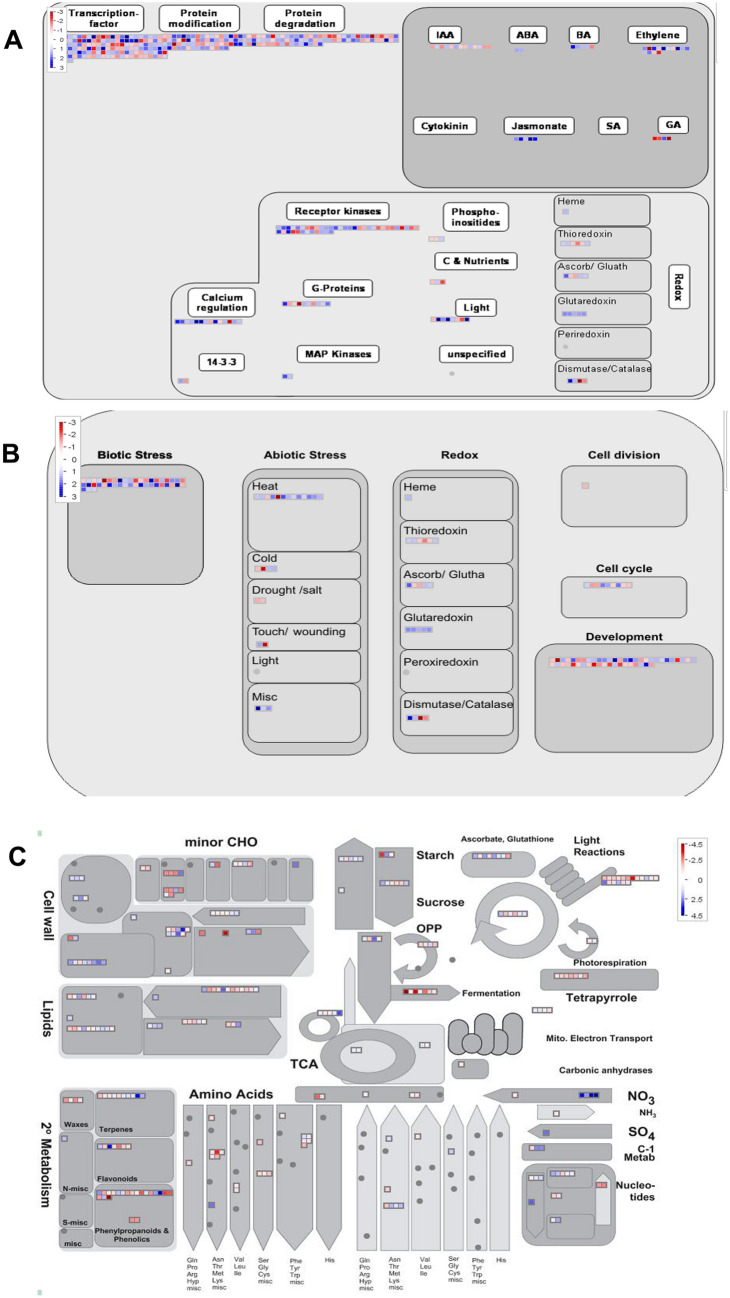
MapMan pathway analysis of *RGA1*-responsive genes. All the DEGs identified in our study were mapped into regulation (**A**), cellular responses (**B**) and metabolic pathways (**C**) using MapMan tool (https://mapman.gabipd.org/mapman). DEGs are represented by either blue or red colour box indicating their up- and down-regulated expression, respectively.

The GO enrichment terms for up- or down-regulated genes are shown separately in Table S1. Response to stimulus was the only over-represented biological process for down-regulated DEGs; however, the up-regulated genes were enriched in many more processes such as, stimulus, biotic and abiotic stress, biosynthetic and metabolic pathways, biological regulation and gene expression among others (Table S1), suggesting their regulation by *RGA1* function. A detailed analysis of the DEGs related to abiotic stress response was described earlier^28^. To further understand the genomewide functions of *RGA1* regulated genes, the DEGs were subjected to in-depth pathway analyses using MapMan^42^. It revealed secondary metabolism, nucleotide metabolism, nutrient and metal transport, in addition to supporting the various categories found in the GO analyses. A number of DEGs were mapped for hormone signaling, protein modification and degradation (Fig. 1A). A majority of the genes regulated by *RGA1* were related to ethylene and jasmonate pathways, but some were also involved in auxin, GA and ABA function (Fig. 1A). A large number of DEGs associated with biotic stress, development and metabolic pathways were also found to be regulated by *RGA1* in rice (Figs. 1B,C) as discussed later.

### *RGA1* regulates genes involved in cell wall, lipid, biotic stress and C/N metabolism

**Figure 2:**
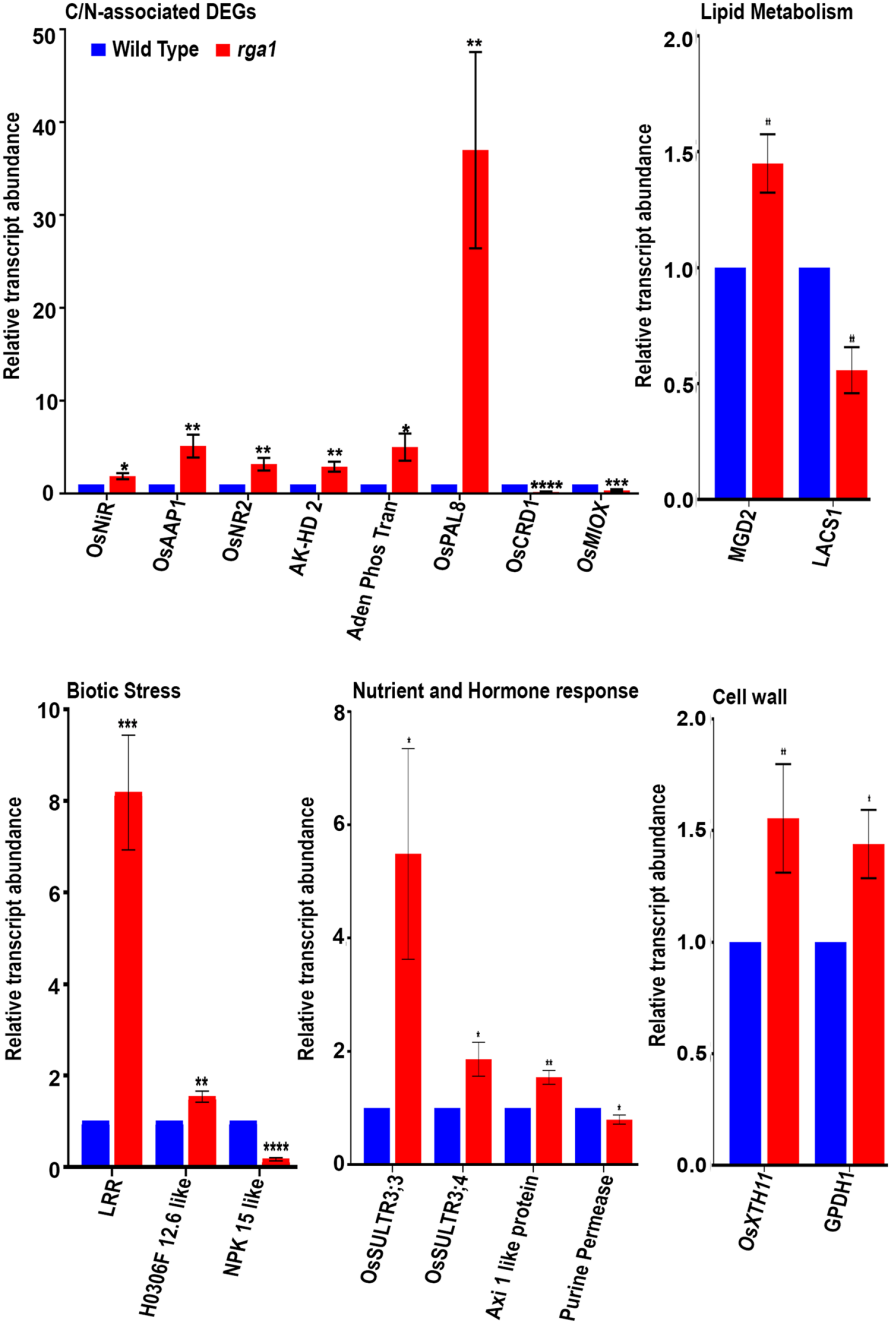
RT-qPCR validation of the regulation of selected DEGs involved in C/N metabolism, lipid metabolism, nutrient and hormone response, biotic stress and cell wall in the *rga1*-mutant. RT-qPCR was performed using total RNA isolated from leaves of the wild type and *rga1* mutant plants grown under conditions similar to microarray analyses for five DEGs, *Aden Phos Tran*, *OsPAL8, AK-HD 2*, *OsXTH11* and *OsMIOX*, using Actin gene (LOC_Os03g50885) as the internal control. To check the expression of additional DEGs at active tillering stage, leaves from 52 day old plants grown under light intensity of 2800 ± 100 lux were used. Their RNAs were used for RT-qPCR on the genes *OsNiR*, *OsAAP1*, *OsNR2*, purine permease, *OsCRD1*, *GPDH1*, *MGD2*, *LACS1*, *LRR*, *H0306F 12.6 like*, *NPK 15 like*, *OsSULTR3;3*, *OsSULTR3;4* and *Axi 1 like protein*, with actin gene (LOC_Os01g64630) gene as an internal control gene for data normalization. The experiments were performed using two biological and three technical replicates. Relative amounts of transcripts are represented with Mean ± SE. Unpaired t-test analyses was performed using the software GraphPad Prism 6.0 (https://www.graphpad.com/scientific-software/prism/). *P < 0.05, **P < 0.01, ***P < 0.001.

The expression profile of *RGA1*-regulated genes on the microarray was validated by RT-qPCR using 19 DEGs chosen based on their roles in important processes but not well known in terms of their regulation by G-proteins in rice (Fig. 2). We validated 6 up- and 2 down-regulated genes of C/N metabolism. The upregulated genes were ferredoxin-nitrite reductase (LOC_Os01g25484, *OsNiRs*), amino acid permease (LOC_Os07g04180, *OsAAP1*), nitrate reductase (LOC_Os08g36500, *OsNR2*), bifunctional aspartokinase/homoserine dehydrogenase (LOC_Os09g12290, *AK-HD 2*), adenine phosphoribosyltransferase (LOC_Os07g30150, *Aden. Phos. Tran.*) and phenylalanine ammonia-lyase (LOC_Os11g48110, *OsPAL8*). The down-regulated genes were magnesium-protoporphyrin IX monomethyl ester cyclase (LOC_Os01g17170, *OsCRD1*) and myo-inositol oxygenase (LOC_Os06g36560, *OsMIOX*). Lipid metabolism gene 1,2-diacylglycerol 3-beta-galactosyltransferase (LOC_Os02g55910, *MGD2*) was up-regulated whereas long-chain-fatty-acid-CoA ligase family protein (LOC_Os05g04170, *LACS1*) was down-regulated in the *rga1* mutant. There were also 2 up- and 1 down-regulated genes involved in biotic stress viz., NBS-LRR disease resistance protein (LOC_Os02g38392, *LRR*), similar to H0306F 12.6 protein (LOC_Os04g58700), and serine/threonine specific protein kinase NPK 15 like l (LOC_Os01g48390), respectively. Three up-regulated genes were involved in nutrient and hormone response, namely, putative sulfate transporters (LOC_Os04g55800, *OsSULTR3;3* and LOC_Os06g05160, *OsSULTR3;4*) and similar to auxin independent growth promoter 1 like protein (LOC_Os09g27080, *Axi like protein*), whereas putative purine permease (LOC_Os04g49757) was down-regulated in the *rga1* mutant. Two of them were up-regulated genes involved in cell wall, glycerol-3-phosphate dehydrogenase (LOC_Os05g41590, *GPDH1*) and xyloglucan endotransglucosylase (LOC_Os06g48160, *OsXTH11*). The primer details used for RT-qPCR are given in Table S2.

### *RGA1* regulates genes involved in important morphological traits

**Figure 3:**
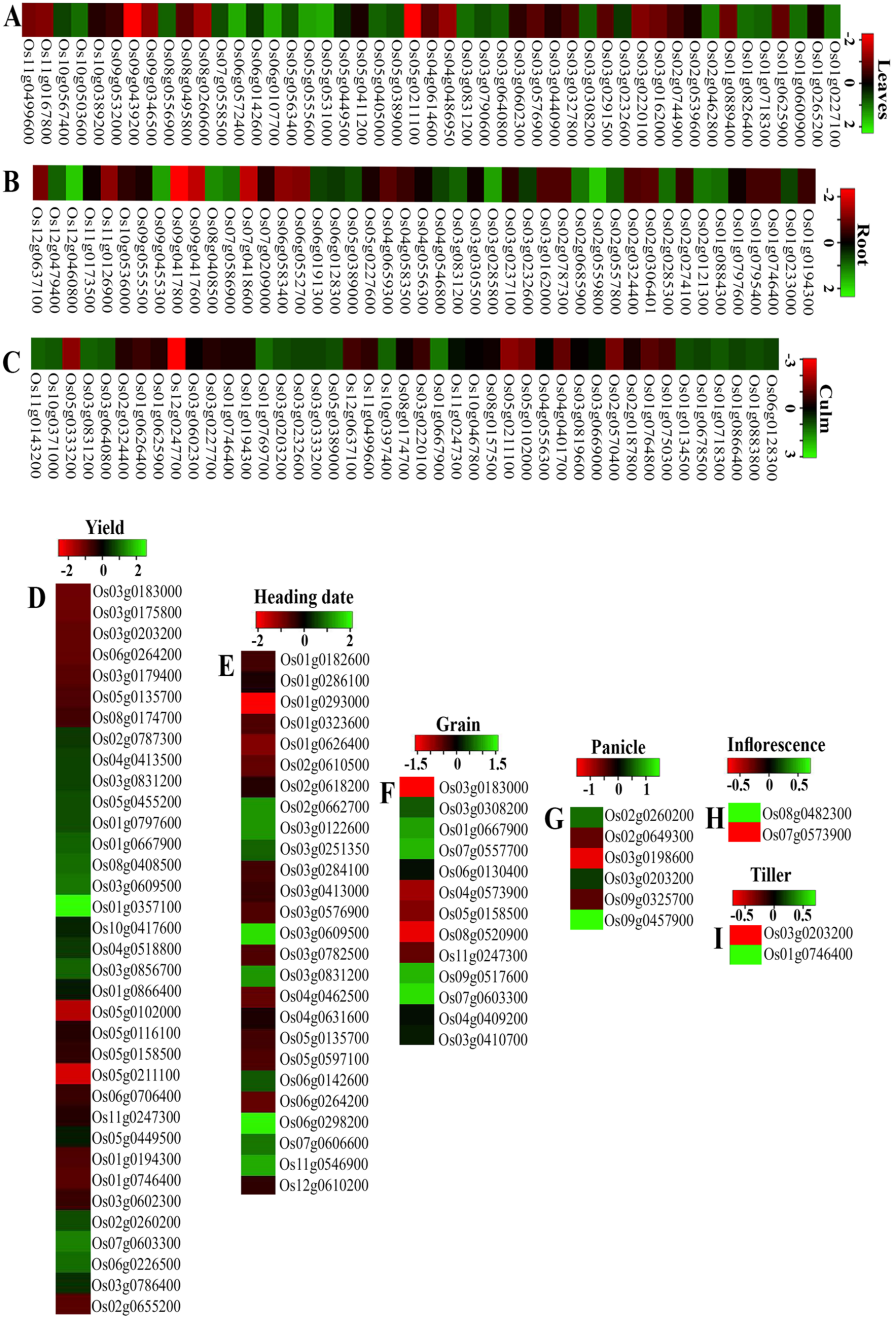
The *RGA1*-regulated gene expression patterns in rice morphological traits. The heat map shows the tissue-specific relative transcript abundance of DEGs identified in rice *rga1* mutants as compared to wild type plants. Heat map was generated using heatmapper (http://heatmapper.ca/). The expression pattern of DEGs with default colour scale is shown for leaves (**A**), root (**B**), culm (**C**), yield (**D**), heading date (**E**), grain (**F**), panicle (**G**), inflorescence (**H**) and tiller (**I**).

Loss of *RGA1* function leads to physiological and phenotypic changes in the *rga1* mutant of rice, mediated by spatio-temporal changes in the expression of many genes. Therefore, it was of interest to examine how many DEGs identified here are expressed in different organs of the rice plant. This allows separating those genes that are ubiquitously expressed in most of the organs from those that are unique to specific organs/stages for further validation of their RGA1/G-protein mediated regulation in rice. To catalogue the trait-associated genes in *rga1* mutant, the DEGs were searched in literature/Oryzabase database and assigned to the various known morphological traits (Fig. 3). A total of 43 DEGs assigned to the culm/plant height showed up-regulation for gibberellin 20 oxidase 2 (Os01g0883800), cytochrome P450 (Os11g0143200), START domain containing protein (Os03g0640800), AP2 domain containing protein (Os05g0389000) and down-regulation for cellulose synthase (Os01g0750300, Os10g0467800), DEAD-box ATP-dependent RNA helicase (Os03g0669000) and potassium transporter (Os04g0401700), among others.

Forty five DEGs were found to be involved in the regulation of root system architecture, of which the up-regulated DEGs are RING-H2 finger protein (Os02g0559800), serine/threonine-protein kinase (Os12g0460800) and ethylene-responsive transcription factor (Os08g0408500), whereas down-regulated include AP2 domain containing protein (Os01g0797600) and expansin precursor (Os04g0583500), among others.

We observed a mixed expression pattern of the DEGs involved in inflorescence/panicle development (Fig. 3). For example, AP2/ERF transcription factor (Os09g0457900) and kelch repeat-containing F-box protein (Os02g0260200) involved in panicle regulation were up-regulated (Fig. 3). On the other hand, alpha/beta hydrolase (Os03g0203200), HD-ZIP I protein (Os02g0649300), homeodomain-leucine zipper transcription factor (Os03g0198600) and protein phosphatase 2C (Os09g0325700) were down-regulated. Considering the reported increase of PP2C during early panicle development^43^, our finding on its down-regulation in the *rga1* mutant explains its phenotype of reduced panicle development.

**Figure 4:**
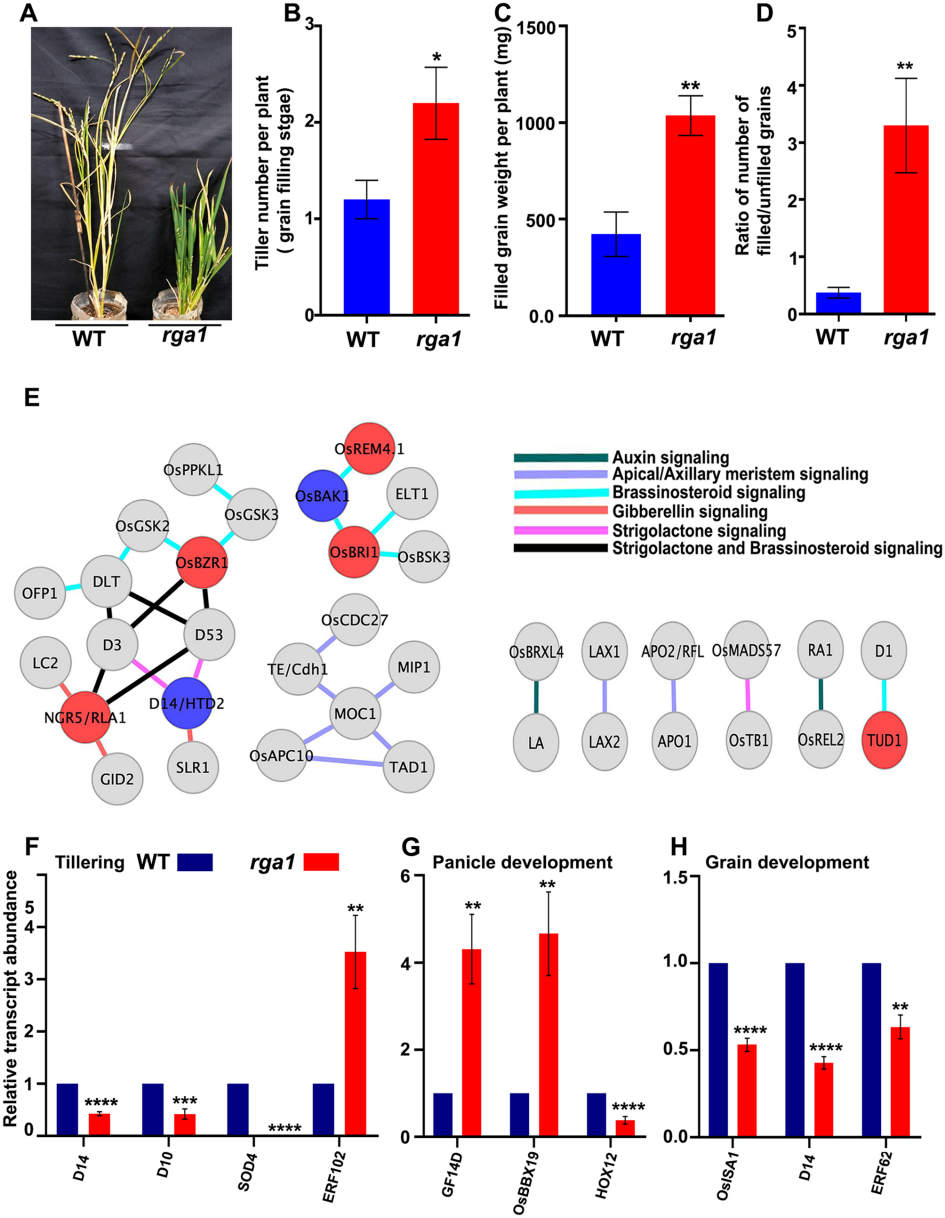
Phenotypic analysis of *rga1* mutant and validation of identified DEGs involved in yield traits. Five plants each were grown in a growth chamber maintained at 25 °C, 70% relative humidity and 12/12 photoperiod with illumination of 2800 ± 100 lux and the trait data were averaged from five plants for tillering and filled grain weight and four plants for the ratio of the number of filled and unfilled grains per plant. (**A**) Image of wild type and *rga1* mutant plants at grain filling stage; (**B**) Total number of tillers per plant at grain filling stage; (**C**) Average filled grain weight per plant; (**D**) Ratio of the number of unfilled and filled grains per plant (**E**) *RGA1*-responsive protein–protein interaction networks of tiller development in rice. Literature-based data were used to retrieve the experimentally validated interactors and networks were constructed using free access software Cytoscape (https://cytoscape.org/). The red and blue nodes represent the up- and down-regulated DEGs in our study and reported earlier^31^, whereas interactors but not DEGs are depicted by grey colour. Edge colors show the type of signaling pathways involved in tiller development. Network details used in Cytoscape are mentioned in Table S3. Expression of genes involved in tiller formation (**F**), panicle development (**G**) and grain development (**H**). Relative transcript abundance was determined using qPCR. Data were normalized using actin as a reference gene and relative fold change was calculated using comparative Ct method. Experiments were performed using two biological and three technical replicates. Statistical unpaired t test analyses were performed on the pooled data (**p value < 0.01, ***p value < 0.001, ****p value < 0.0001). *D14* (LOC_Os03g10620, alpha/beta hydrolase family), *D10* (LOC_Os01g54270, transposon protein), *SOD4* (LOC_Os08g44770, Cu/Zn-SOD), *ERF102* (LOC_Os09g28440, AP2 domain containing protein), *GF14D* (LOC_Os11g34450, 14-3-3 protein), *OsBBX19* (LOC_Os06g19444, CCT/B-box zinc finger protein), *HOX12* (LOC_Os03g10210, homeobox domain containing protein), Os*ISA1* (LOC_Os08g40930, alpha amylase), *ERF62* (LOC_Os03g08470, AP2 domain containing protein).

We found that while the WT plants germinated earlier and grew faster than the *rga1* mutant leading to early initiation of tillers and heading, the mutant plants performed better in 3 crucial yield parameters viz. number of tillers, total weight of filled grains per plant and the ratio of filled to unfilled grains (Fig. 4) in our experimental growth conditions. Wild type plant showed early emergence of the first tiller as compared to mutant plants, but we observed relatively higher number of branches/tillers in mutant plants over the complete life cycle. Even though the total weight of filled grains per panicle was higher in the wild type, the *rga1* mutant had more panicles and therefore better grain yield (Fig. 4). These results were observed throughout the life cycles of wild type and *rga1* mutants, which is in line with the earlier reports^29,30^. To understand the role of RGA1 in the tiller development, we developed protein–protein interaction networks using experimentally validated interactors reported in tillering phenotypes in rice. We observed sub-networks as well as binary interactions (Fig. 4E), which revealed many proteins involved in the development of apical and axillary meristems and hormone pathways, especially brassinosteroid, strigolactone, gibberellin and auxin in rice. Mapping of DEGs and their expression profile onto the networks revealed that many well characterized interactors such as NGR5/RLA1, OsBZR1, OsBRI1, OsREM4.1 were up-regulated in *rga1* mutant whereas down-regulated DEGs were D14/HTD2 and OsBAK1, indicating the RGA1/G-protein mediated regulation of tiller development in rice.

To explore the molecular basis of the phenotypic differences for these traits, the DEGs associated with tiller, panicle and grain development in rice were retrieved from microarray datasets and literature search. Among them, we validated four *RGA1*-responsive genes involved in tiller formation in the mutant. They were, *D14* (LOC_Os03g10620), *D10* (LOC_Os01g54270), *OsSOD4* (LOC_Os08g44770) and *OsERF102* (LOC_Os09g28440). Those involved in panicle development were *Os14-3-3* (LOC_Os11g34450), *OsBBX19* (LOC_Os06g19444) and *OsHox12* (LOC_Os03g10210). The genes involved in grain development were *OsISA1* (LOC_Os08g40930), *D14* (LOC_Os03g10620) and *OsAP2-125* (LOC_Os03g08470). RT-qPCR experiments validated *RGA1*-regulation of 4 genes related to tiller development and 3 each related to panicle and grain development, including 1 gene common to tiller and grain development (Fig. 4E). These results indicate an important role for *RGA1* in the regulation of tiller development and grain yield in rice.

### *RGA1* modulates genes involved in cell wall composition

As the major constituents of plant cell wall are carbohydrates, we examined the 85 DEGs associated with cellular carbohydrate metabolic process. Among them, 45 genes were significantly up- regulated and 40 genes were down-regulated by *RGA1* mutation (Table S4). Among them, there were genes involved in cell wall component synthesis and cell wall remodeling in rice. For instance, cellulose synthase-6 (Os03g0837100), sucrose synthase 2 (Os03g0401300) and starch synthase (Os01g0720600) genes were up-regulated by 1.5, 1.7 and 1.1 fold, respectively. We also detected 1.4 and 3.3 fold down-regulation of glycosyl transferase (Os05g0552200) and 1,4-alpha-D-glucan glucanohydrolase (Os08g0473900) genes, respectively in the *rga1* mutant. These results showed that the loss of *RGA1* function significantly changes the expression of genes involved in carbon metabolic pathways, including those that contribute to cell wall synthesis in rice. Further, Toluidine blue permeability test on seeds revealed the enhanced permeability of cuticle in *rga1* mutants compared to the wild type, indicating altered cell wall composition in the *rga1* mutants (Fig. S3).

### *RGA1*-dependent lipid transport and metabolic regulation

Sixty seven DEGs in the *rga1* mutant were found to be associated with lipid transport and metabolism (Table S4). Lipid transfer proteins (LTPs) play important roles in defense response and also regulate flower and seed development^44,45^. Most of the DEGs identified in this category are down-regulated, such as lipid transfer protein LPT III (Os11g0427800), lipid transfer protein and hydrophobic protein (Os12g0115300 and Os10g0554800), nonspecific lipid-transfer protein 1 and 3 (Os11g0116000, Os12g0115500) (Table S4), whereas DEGs associated with lipid metabolism showed mixed expression patterns, suggesting that *RGA1* regulates these pathways in plants.

### *RGA1* regulates N-response pathways

In view of our earlier findings on the role of G-proteins in nitrate reductase gene expression in maize^25^ and rice^35^, as well as its association with N-use efficiency (NUE) in rice^46^, we examined the expression of N-regulated genes in the *rga1* mutant. We found 53 DEGs to be associated with cellular nitrogen compound metabolic process (Table S4). We found the up-regulated expression of nitrate reductases (Os08g0468100 and Os08g0468700), ferredoxin-nitrite reductase (Os01g0357100), CC-type glutaredoxin (Os01g0667900), CTP synthetase 1 (Os05g0573300), nitrate-inducible and auto repressible transcriptional repressor (Os02g0325600) and BT1/BT2 ortholog (Os01g0908200) among others in the *rga1* mutant. Interestingly, the down-regulated genes include ammonium transporter (Os05g0468700), nitrate transporter (Os10g0554200), alanine aminotransferase 2 (Os10g0390600) and glutamate dehydrogenase (Os03g0794500) highlighting the importance of *RGA1* function in N-uptake, translocation, signaling and possibly contributes to NUE.

**Figure 5:**
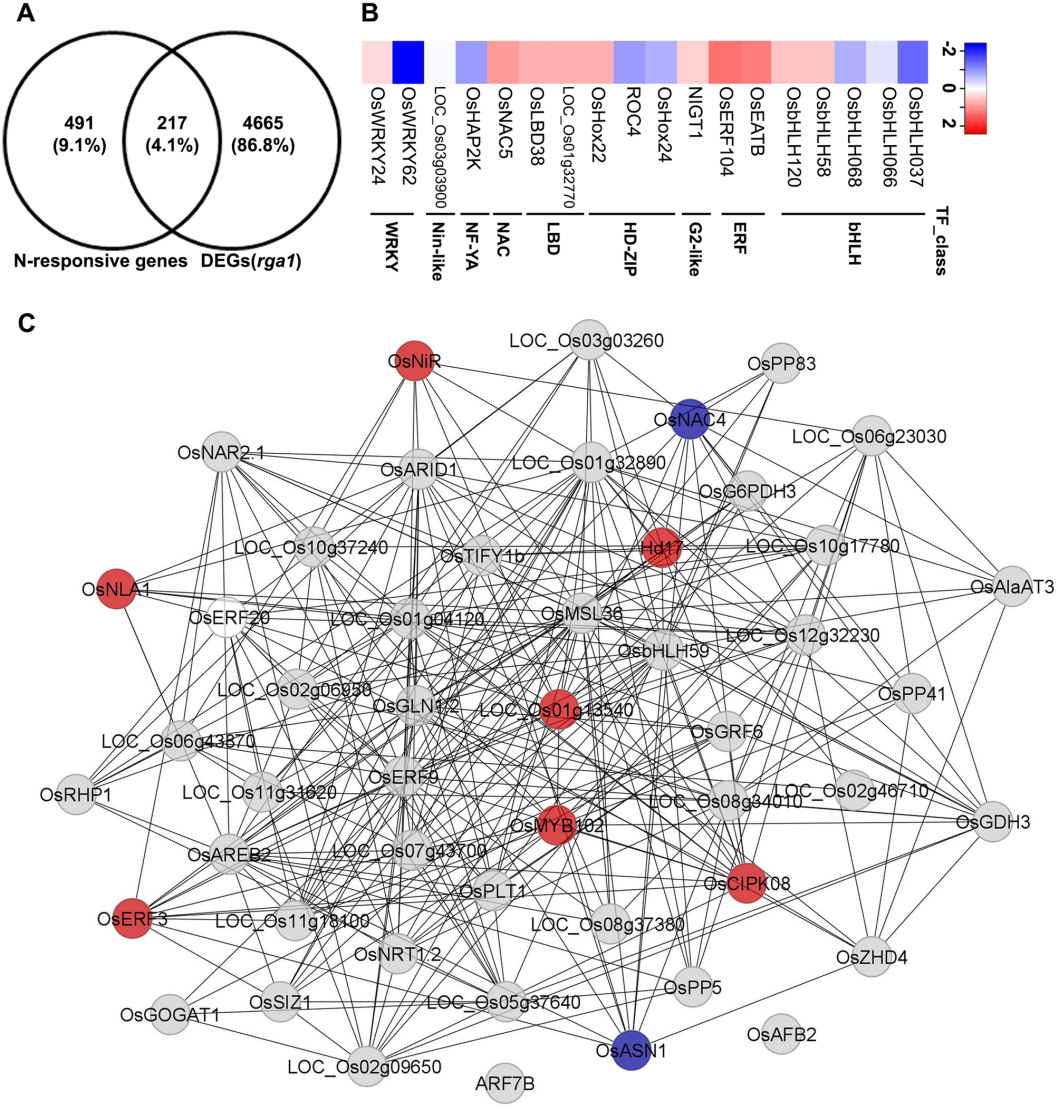
RGA1-regulated N-responsive transcriptional regulatory network (TRN) in rice. (**A**) Venn diagram depicting the DEGs showing commonality with N-responsive transcriptomes known in rice^47–52^. (**B**) Heat map showing the expression profile of N-responsive transcription factors identified as DEGs in the *rga1* mutant. Heatmapper (http://heatmapper.ca/) was used to generate the heat map and z-scores correspond to fold change value (log_2_FC) of DEGs representing the colour on scale bar. (**C**) TRN involved in nitrate signaling was constructed in Cytoscape version 8.0.0 (https://cytoscape.org/) based on nitrate-regulated TRN available in Arabidopsis^53^. Expression profile of DEGs identified in this study and reported earlier^31^ was mapped onto the network. The red and blue nodes depict the up- and down-regulated DEGs identified in the *rga1* mutant whereas light grey colour nodes are not DEGs. Network constituting fifty highly interconnected nodes are displayed and complete *RGA1*-regulated nitrate-responsive TRN is provided in Fig. S5.

To further delineate the *RGA1* function in N-response and signaling, DEGs identified in this and earlier study [31] were searched in N-responsive transcriptomic data available in rice^47–52^. To identify the genes involved in N-responses and signaling, the DEGs were subjected to venn analysis, which showed 217 DEGs associated with N-transport, -metabolism, -signaling and -transcriptional regulation, among others (Fig. 5A and Table S5). Transcriptional Factors (TFs) and their target genes that constitute the transcriptional regulatory network (TRN) for RGA1/G-protein involvement in N-responses is not known in plants. *RGA1*-regulated TFs were identified by searching all the DEGs in RGAP database (http://rice.plantbiology.msu.edu/), which revealed 297 TFs belong to 40 families (Table S6) in rice. Among these, bHLH, ERF, WRKY, MYB and NAC were the most abundant TF families that showed differential regulation in *rga1* mutant (Fig. S4). These identified TFs were further searched in literature and the N-responsive transcriptomes^47–52^, which revealed 18 TFs as N-responsive in *rga1* mutant (Table S7). Transcript accumulation pattern of these 18 N-responsive TFs is shown as a heat map (Fig. 5B). Nitrate-regulated TRN is not well established in rice, whereas such network was developed and validated using knockout mutants in Arabidopsis^53^. Using Arabidopsis orthologous information, similar nitrate-regulated TRN was constructed in rice. We retrieved all the TRN interactors from Arabidopsis, identified their corresponding orthologs in rice and used them to construct TRN in Cytoscape. Expression profile of DEGs identified in *rga1* mutant were mapped onto constructed network in Cytoscape. Filtering was applied in Cytoscape to display 50 highly interconnected nodes (Fig. 5C), whereas complete network is shown in Fig. S5 and associated information in Table S8. We observed that loss of function of *RGA1* perturbed the expression of N-regulated TFs such as Nin like, WRKY and bZIP among others and well known target genes such nitrite reductase, glutamine synthetase, OsCIPK23, urea transporter (OsDUR3), indicating the key role of *RGA1* in N-response and N-signaling in rice (Fig. 5, Fig. S5).

### Regulation of hormone biosynthesis and signaling in *rga1* mutant

Developmental as well as defense responses are known to be regulated by signals triggered by hormones such as auxin (AUX), gibberellin (GA), cytokinin (CK), ethylene (ET), jasmonic acid (JA) and brassinosteroid (BR) in plants^54^. In the present study, most genes related to these hormones showed mixed expression patterns in the *rga1* mutant (Table S4). Importantly, we observed the up-regulated expression of transporter (Os04g0459000) involved in auxin transport whereas mixed expression for auxin-responsive SAUR gene family (Os02g0769100, Os09g0545300, Os06g0714300, Os04g0662400) in the *rga1* mutant. The BR biosynthesis genes (Os07g0580500, Os10g0397400) and brassinosteroid signaling multi-tasking co-receptor BAK1 gene (Os05g0414700) were induced, whereas reduced accumulation was observed for associated transcription factor (Os03g0169600).

### *RGA1* regulates biotic stress responses

A large number of DEGs that are functionally annotated to “response to stimulus” and “response to stress” categories were found to be associated with both biotic and abiotic stress responses (Fig. 1B). As we already reported the DEGs related to abiotic stress response^28^, only those related to biotic stress are elaborated here. We detected the down-regulation of many DEGs including respiratory burst oxidase homolog D (*RBOHD*, LOC_Os01g25820), chitinase (*POM1*, LOC_Os08g41100), and reticulon family protein (LOC_Os07g04910) among others (Table S4). The up-regulated DEGs were harpin-induced protein 1 domain containing protein (LOC_Os04g58850), target of AVRB operation 1 (*TAO1*, LOC_Os02g38392) pathogenesis-related thaumatin family protein (LOC_Os10g05600), resistance to *Pseudomonas syringae* pv. maculicola 1 (*RPM1*, LOC_Os08g07774), mildew resistance locus O 12 (*MLO12*, LOC_Os03g03700) among others (Table S4). The MLO protein was implemented in the regulation of immunity involving G-protein complexes in Arabidopsis^55^. The sphingolipid elicitor of *Magnaporthe oryzae* activates the OsMAPK6 pathway and regulates the expression of phenyl ammonia lyase and PR proteins during pathogenesis in *rga1* mutants^32^. Our microarray data revealed the differential regulation of receptor like kinases (RLKs), phenyl ammonia lyase and PR genes in *rga1* mutants (Table S4). The up-regulation of phenyl ammonia lyase in the *rga1* mutant was confirmed by RT-qPCR (Fig. 2). Together, these findings indicate important and hitherto unknown roles for *RGA1* in fine tuning of biotic stress responses, which are yet to be characterized.

### *RGA1* regulates submergence responses in rice

**Figure 6:**
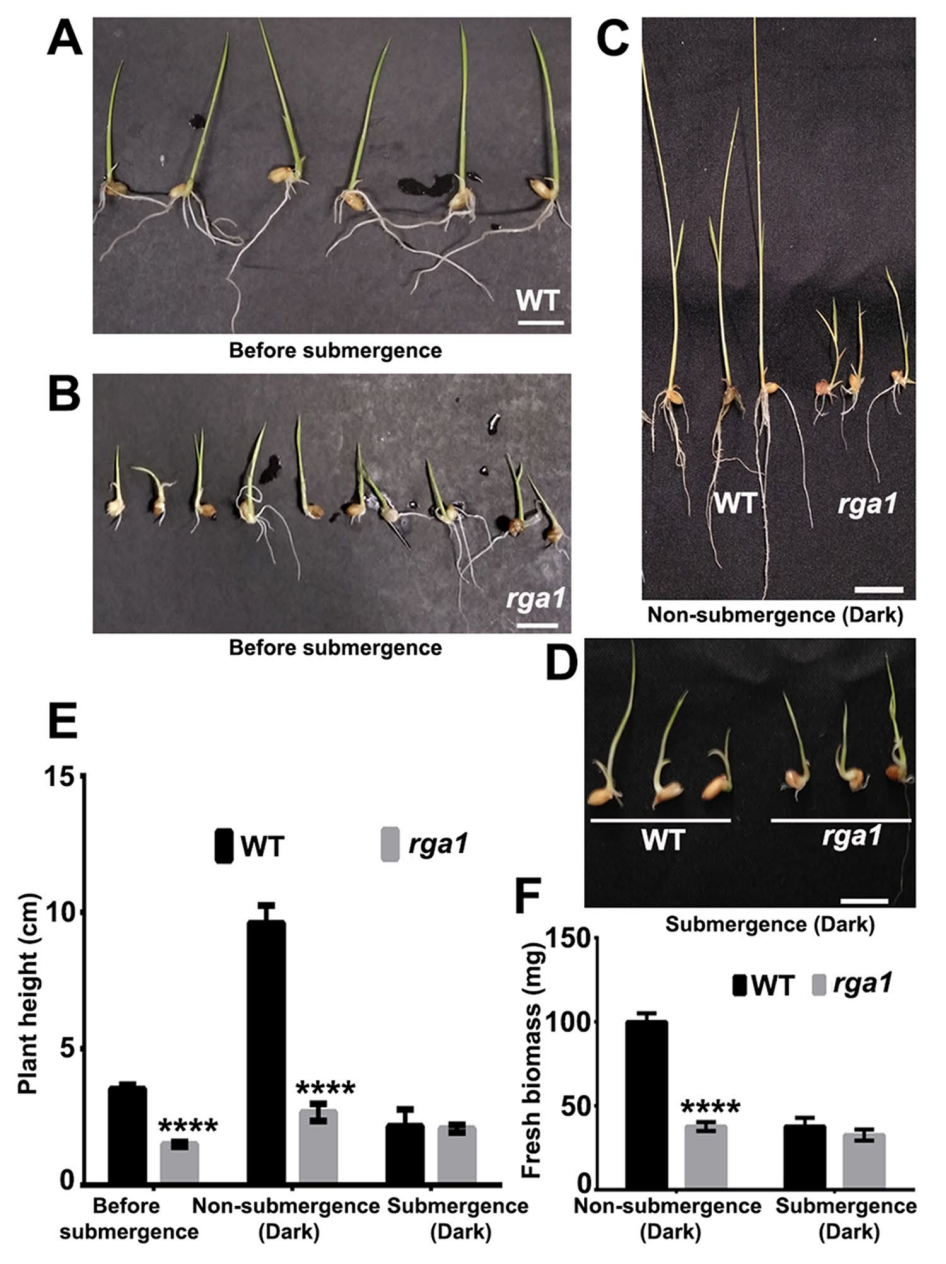
Mutation in *rga1* protects rice from reduced growth under submergence stress. Representative image of wild type (**A**) and *rga1* mutant (**B**) seedlings before submergence, non-submergence control (**C**) and submergence (**D**) in the dark. Plant height (**E**) and fresh biomass (**F**) are severely affected by submergence in the wild type, but not in the *rga1* mutant seedlings, relative to their with non-submergence controls. Data represent average of at least 3 seedlings ± SE. Scale bar = 1.0 cm. Unpaired t-test was performed using the GraphPad Prism 6.0 (https://www.graphpad.com/scientific-software/prism/). ****p value < 0.0001.

Heterotrimeric Gα subunit is involved in abiotic stress responses in Arabidopsis^40^ and rice^28^. In this study, we measured the effect of submergence on the *rga1* mutant in dark condition. Before submergence, *rga1* mutant seedlings had significantly lower plant height (*p* < 0.0001) relative to the wild type (Fig. 6A,B,E). After submergence mutant seedlings displayed no significant effect on plant height while that of the wild type was drastically affected by submergence (Fig. 6C–E). Further, the fresh biomass of the mutant seedlings was not significantly affected by submergence whereas that of the wild type was drastically affected (Fig. 6F), clearly indicating that submergence does not impact the growth of *rga1* mutant. In other words, *rga1* mutation protects the plant from the adverse effect of submergence stress on plant growth in the dark.

### Protein–protein interaction networks reveal RGA1 role in metabolic process, stress responses and gene regulation

**Figure 7:**
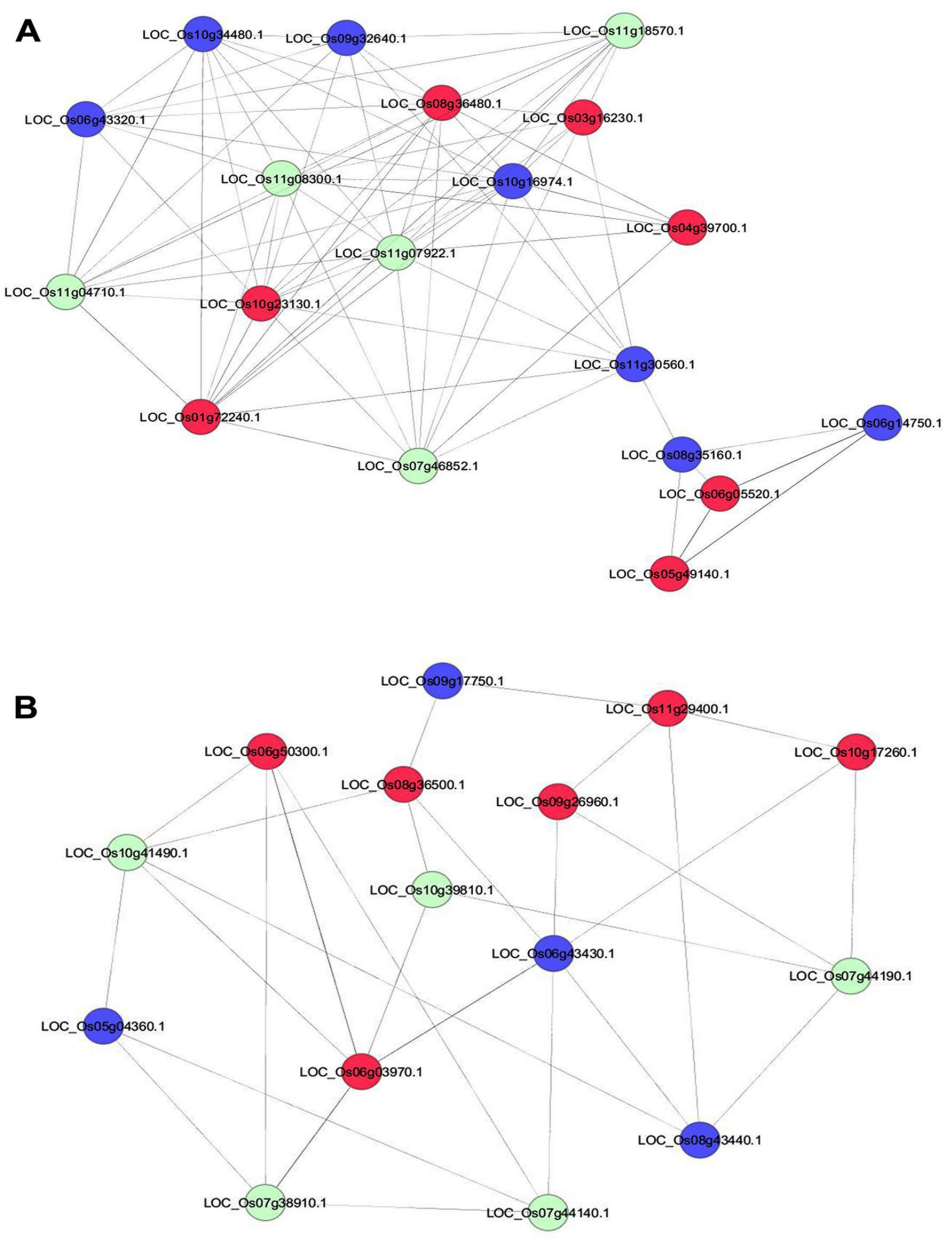
RGA1-regulated molecular complexes associated with different cellular pathways. All the DEGs identified in our study were used to retrieve the experimentally validated interactors from BioGRID, STRING and other databases. The protein–protein interaction (PPI) networks were constructed using DEGs-associated interactors in Cytoscape and molecular complexes were identified using the MCODE plugin in Cytoscape. Representative RGA1-regulated molecular complexes are shown (**A** and **B**) and the rest of the identified molecular complexes are given in Fig. S6–S11. The red and blue nodes represent the up- and down-regulated DEGs, respectively. Interactors that are not DEGs are assigned with light colour.

To understand the cellular function of RGA1, we developed protein–protein interaction (PPI) networks and mapped the identified RGA1-regulated DEGs into the networks. We retrieved the experimentally verified interaction data from STRING, MCDRP, BioGRID and PRIN databases and constructed the networks where DEGs expression value were used to colour-code the nodes. The networks were analyzed and viewed in network visualization software Cytoscape 8.0.0^56^. The network consisted of 1058 nodes and 4776 edges after removing the duplicated edges in Cytoscape. We observed that the DEGs formed a majority of the interacting proteins in the network. To identify the interacting molecular complexes for better clarity, MCODE analyses were performed and eight highly connected sub-clusters were identified. We have selected four sub-clusters having MCODE score > 3 with node number > 10 (Fig. 7 and Figs. S6–S9). A total of 19, 15, 23, 37 nodes and 78, 29, 40, 61 edges were detected in sub-cluster 1, 2, 3 and 4, respectively. The details of all eight sub-clusters are provided in Table S9. We further performed the GO enrichment analysis of the genes in these sub-clusters by AgriGO (Table S9). The GO analyses showed that genes in sub-cluster 1 are mainly associated with “metabolic process” and in sub-cluster 2 with “response to abiotic stimulus” along with “metabolic process”. The genes in sub-cluster 4 was associated with “response to stress” and “response to abiotic stimulus” whereas in sub-cluster 3 enrichment for various biological processes was “cellular protein metabolic process”, “cellular macromolecule metabolic process”, “cellular biosynthetic process” “gene expression” “response to biotic stimulus” among others (Table S9). The preponderance of DEGs in the top sub-clusters indicates that RGA1 regulation may be primarily mediated by these sub-clusters.

A comparison of the 2270 DEGs identified in our genome-wide microarray analyses with the 3327 DEGs reported in a recent RNA-seq analysis of the impact of *RGA1* mutation^31^ revealed that the number of exclusive DEGs was far higher than the common DEGs (Fig. S12A). The overlapping DEGs were only 537, whereas 1556 DEGs are exclusive to our study and 2790 others were exclusive to the other study^31^. To understand the reasons for higher number of exclusive DEGs detected in both the studies, we performed Venn analyses between non-redundant transcripts corresponding to 30205 unique probes spotted on 4 × 44 K rice microarray used in this study and DEGs identified by RNA-seq elsewhere^31^.Venn analyses clearly showed that 3236 DEGs out of 3397 DEGs identified by RNA-seq were spotted on the microarray chips and only 161 DEGs were not spotted (Fig. S12B). This indicates both platforms are largely comparable in terms of the coverage of the rice transcriptome. However, the reasons for exclusive DEGs in both the studies could be related to different growth conditions including different light intensities, media and especially different plant age for transcriptomics, which was 25 days in our study whereas 100 days in other^31^.

For a comprehensive and unambiguous comparison of both the transcriptomic data, we converted DEGs to RGAP IDs and carried out sub-cellular prediction by CELLO program and GO analyses using AgriGO tool. The percentage of total DEGs identified in both the studies showed similar sub-cellular distribution with majority of DEGs associated with the nucleus, followed by chloroplast, cytoplasm and plasma membrane, among others (Fig. S12C). However, due to the existence of exclusive DEGs in both the studies, GO enrichment analyses revealed many exclusive pathways in addition to the overlapping ones. A closer examination revealed a higher percentage of DEGs associated with metabolic process, response to stimulus and response to stress in our study with the overlapping DEGs (Fig. S12D), further confirming the broad categories of processes regulated by *RGA1* as identified from our own microarray data.

We further developed the PPI networks using all the retrieved interactors associated with exclusive DEGs identified in both the studies. A total of 451 exclusive DEGs-associated interactors were identified in this study, whereas 1274 interactors were detected in the other study (Fig. S13). We did not find any significant overlap between these interactors (Fig. S13), presumably due to the exclusive DEGs involved in both studies. Further, GO based functional classification also revealed the exclusiveness in the top enriched GO terms such as “DNA conformation change”, “protein-DNA complex assembly”, “nucleosome organization”, “chromatin assembly”, “nucleosome assembly”, “DNA packaging” in our study and “translation” “cellular protein metabolic process” “gene expression” “small molecule metabolic process” “protein metabolic process” “protein folding” in the other study (Table S10).

### Rice G-protein interaction networks

**Figure 8:**
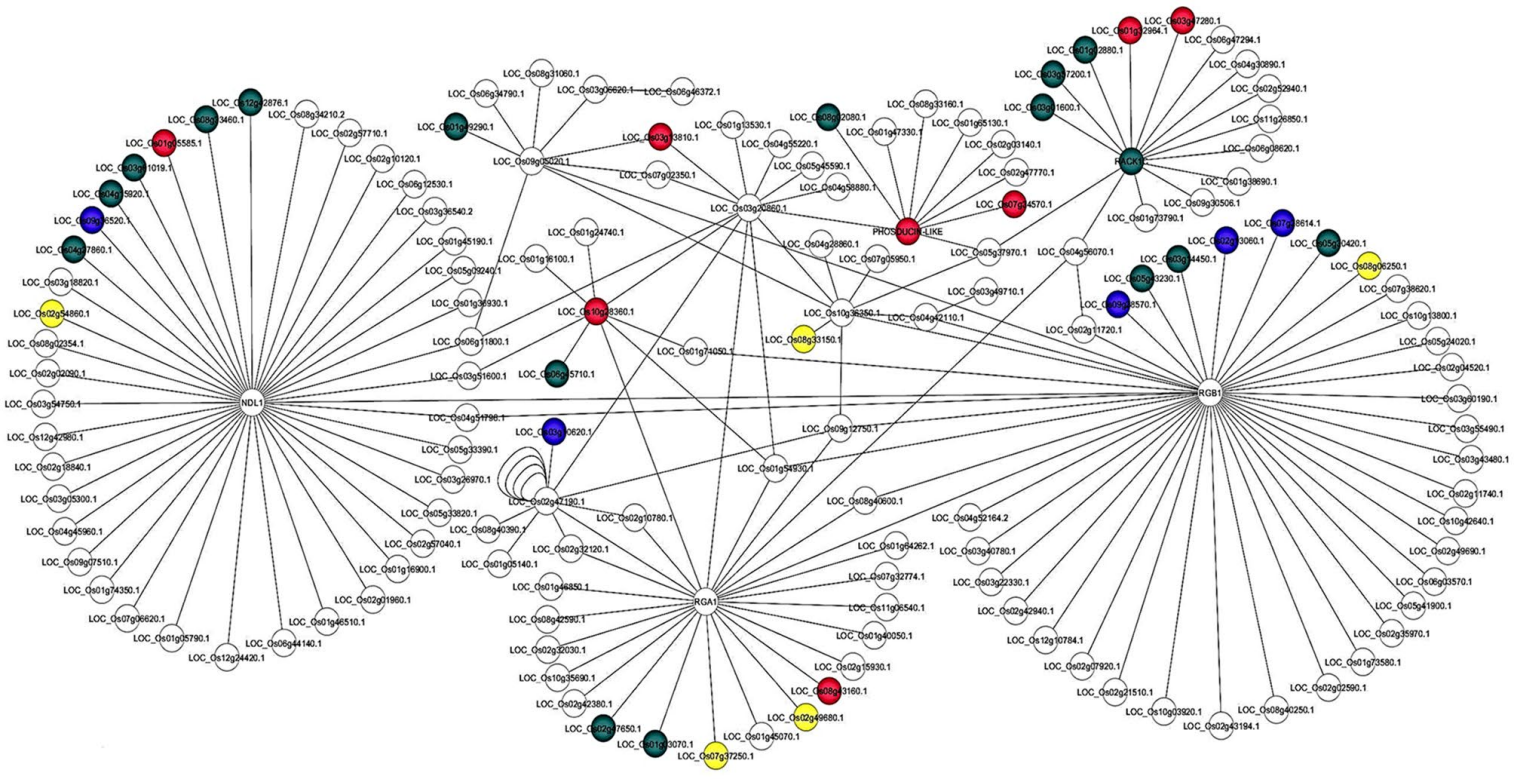
Rice heterotrimeric G-protein interactome and mapping of the expression profile of *RGA1*-regulated genes. Rice G-protein interactome was constructed using orthologs from published Arabidopsis G-protein network^57^. It revealed many well-known and experimentally validated core G-protein interacting hubs in rice. We also mapped the *RGA1*-regulated DEGs identified in our study, and those published earlier^31^. The red and blue nodes represent the up- and down-regulated DEGs in our study, whereas and yellow and green depicts up- and down-regulated DEGs in previous study^31^.

Considering that heterotrimeric G-protein signaling is one of the most evolutionary conserved signal transduction pathways in eukaryotes, we constructed ortholog-based G-protein signaling networks in rice (Fig. 8) using high throughput yeast two-hybrid (Y2H) data from Arabidopsis^57^. We downloaded all the rice orthologs from PlantGDB database (http://www.plantgdb.org/), which were generated from the known Arabidopsis orthologs using OrthoMCL program. Using them, we constructed PPI networks consisting of G-protein signaling components. We found 264 conserved orthologs in rice associated with Arabidopsis heterotrimeric G-protein signaling components (Table S11), suggesting their key role in various cellular processes. To check the validity of the constructed G-protein networks in rice (Fig. 8), we analyzed various clusters for different protein complexes and their association with biological processes. We observed well-conserved proteins such as RGA1, G-protein beta subunit (RGB1), thylakoid formation 1 (THF1), N-MYC down-regulated-like protein 1 (NDL1) and receptor for activated C kinase 1 (RACK1) among others, as highly connected clusters (Fig. 8). To identify the over-representation of various GO categories, we have subjected all the identified interactors of G-protein network to AgriGO analyses. The most over-represented GO term for biological process was ‘stimulus response’ common to rice and Arabidopsis, whereas additional GO terms were observed for the Arabidopsis G-protein network (Table S12). This could be due to the absence of well-defined orthologs, as we could not find all the orthologs of Arabidopsis G-protein networks in rice (Table S11). We mapped the DEGs identified in this study as well as by others^31^ onto rice G-proteins networks (Fig. 8). We found that disruption of RGA1 function affects the expression of many G-protein signaling candidates, which either directly or indirectly interact with RGA1 through the formation of molecular complexes. Literature and database search revealed that 43 interaction pairs have been experimentally validated in rice (Table S13), which highlights the quality of G-protein networks in rice. The well-established direct interactors of RGA1 include RGB1 and THF1, whereas indirect interactors are NDL1, RACK1 among others, which form highly connected networks and suggest the diverse functions of RGA1 in rice beyond those reported so far.

## Discussion

We used transcriptome analyses of Arabidopsis mutants earlier to understand the genomewide role of G-proteins^5,6,40,58^ as a dicot model, but the only known Gα mutant in rice produces a more distinct phenotype, which shows weak developmental and environmental responses^17,18^. Moreover, genome-wide analyses in rice as a model monocot and a model crop can provide better insights into the roles of *RGA1*, especially considering its tremendous agronomic value. The only available transcriptomic RNA-seq study on rice Gα subunit mutant was specifically focused on photoprotection and photoavoidance^31^. Our genome-wide microarray data on the *RGA1* mutant in rice were used earlier to analyze a subset of the *RGA1*-responsive genes involved in abiotic stress response^28^. In the present study, we elaborate on the wider roles of *RGA1* through our comprehensive transcriptomic data and network analyses to provide several hitherto unknown insights, including regulation of agronomically important traits and their underlying processes.

We identified 2270 DEGs in the *rga1* mutant and their GO and MapMan analyses revealed that they may impact developmental and stress responses. A number of direct interactors of Arabidopsis G-protein complexes have been identified in literature^57^, but not in rice. Moreover, the absence of regulators of G-protein signaling (RGS1) in rice^10^ makes the RGS1-dependent paradigms advanced in Arabidopsis^9^ irrelevant to rice and necessitates alternative paradigms on rice G-protein signaling.

Our functional annotation analysis shows that a number of DEGs are involved in carbohydrate metabolism, development and biotic stress. A possible common link between them is that carbohydrates are the primary constituents of the plant cell wall, which plays important roles in growth and developmental processes and acts as a frontline defense barrier in plants. We detected the differential expression of many cell wall-associated genes, including the RT-qPCR validated xyloglucan endotransglycosylase and NAD or NADH binding/glycerol-3-phosphate dehydrogenase (Fig. 2). Others include COB-like genes (COBLs), an expansin family member, polygalacturonase, pectinesterase, glycosyl hydrolase family 3 protein, UDP-xylose synthase 4, HAD-superfamily hydrolase, a sucrose synthase and a cellulose synthase among others. COBLs are involved in cellulose synthesis in primary as well as secondary cell wall^59^. Expansins are well known for their role in cell wall expansion, whereas cellulose synthase regulates cellulose composition and development of stem height, leaf and root^60^. Many DEGs found in our study belong to these families and are known to interact with different G-protein signaling components^50^. This suggests their contribution to the growth physiology underlying the morphological/phenotypic characteristics of *rga1* mutants. Further, the higher permeability of the seed cuticle in the *rga1* mutant relative to wild type as revealed by toluidine blue test (Fig. S3) indicates altered cell wall characteristics in the *rga1* mutant that merit further evaluation.

The regulation of G-protein mediated signaling in animals is known to involve complex interactions between lipids and proteins^61^, including the myristoylation and palmitoylation of the Gα protein^62^. We found that DEGs belonging to lipid transfer protein family are highly expressed in flower, shoot and leaves, where they regulate various cellular events^63,64^. It has been demonstrated that *rga1* mutant shows significantly reduced hypersensitive response (HR) to rice blast fungus^32^. This may be due to the loss of coordinated expression of LTP genes in the *rga1* mutant, affecting pathogen sensitivity and developmental phenotypes. We validated the regulation of two DEGs involved in lipid pathways in rice namely, 1,2-diacylglycerol 3-beta-galactosyltransferase (*MGD2)* and long-chain-fatty-acid-CoA ligase family protein (*LACS1*, LOC_Os05g04170) (Fig. 2).

The indispensible roles of reactive nitrogen (N) compounds as nutrients and signals to regulate developmental plasticity, productivity and stress responses are known^65–67^. But their role in the improvement of N use efficiency (NUE) for sustainable agriculture is unknown, as how N-response translates into NUE is still not well understood^68–70^. Our earlier genome-wide studies on Arabidopsis Gα mutant suggested its role in the regulation of N response^5,6^. Earlier pharmacological studies suggested a role for G-protein in the regulation of N-responsive gene expression in maize^25^ and rice^35^. More recently, genetic analyses identified the role of a rice G-protein gamma subunit in NUE^46^. Our analysis here shows for the first time that a number of genes involved in N-uptake, translocation and amino acid metabolism are regulated by *RGA1* in rice, corroborating our earlier findings in Arabidopsis^5,6^ for the first time in any crop plant. Both G-protein signaling and N-responsive signaling are separately known to use TFs to regulate the expression of target genes^71,72^. But the role of G-protein signaling in N-responsive transcriptional regulation is not established in any plant so far. To explore this, we identified N-responsive TFs and developed associated TRN in the *rga1* mutant. We observed the differential regulation of TFs such as WRKY, Nin-like and NAC among others that are well-known in N-response and signaling in rice and Arabidopsis. Further, we constructed TRN based on the nitrate-regulated TRN known in Arabidopsis^53^. We observed similar nitrate-regulated TRN in rice as reported in Arabidopsis, based on currently known orthologs. Though the core of nitrate-regulated genes showed conservation between rice and Arabidopsis, many associated TFs and target genes need validation by genetic and biochemical studies in rice. Our results on the differential regulation of important N-regulated TFs and target genes involved in N-transport, -assimilation and -signaling showed that *RGA1* mediates or regulates N-response and associated signaling rice. The *rga1* mutant showed differential root phenotypes in response to different nitrate doses^73^. However, most of the TFs and their targets are not characterized in terms of their Gα/G-protein-mediated N-regulation, indicating an important area that needs immense investigation. Our RT-qPCR validation of 8 *rga1*-responsive genes of C/N-metabolism further strengthens the role of heterotrimeric G-protein pathways in the regulation of several aspects of N-response and possibly NUE in rice**,** especially considering that its QTL constitutes a G-gamma subunit^46^.

Studies on *RGA1* promoter-driven gene expression showed maximum *RGA1* expression in developing organs and varied with their developmental stages^74^. The genes involved in various morphological traits of *rga1* mutant such as plant height and culm length, small panicles, round grains and small root architecture are yet to be investigated, except for dark-green leaves^31^. The mechanism regulating shoot branching or tillering is better known in dicots as compared to monocots^75^, even though it is an important QTL for yield and NUE in rice. Tillering is controlled by genes involved in meristems, hormones, nutrients and environmental interactions^76^, but its G-protein-dependent regulation is not known. To address this, we constructed experimentally validated protein–protein interaction networks regulating tiller development in rice (Fig. 4E). Gain- or loss-of-function of these interactors were known to alter tillering phenotypes in rice (Table S3). We found many *RGA1*-responsive genes such as NGR5/RLA1, OsBZR1, OsBRI1, OsBAK1 and D14/HTD2 in these networks, indicating the role of G-protein in regulating tiller development in rice. Though we observed crosstalk between brassinosteroid, strigolactone and gibberellin signaling pathways, there was a preponderance of interactors belonging to brassinosteroid signaling pathways (Fig. 4E). Our network revealed the interaction of RGA1 with TUD1, an U-box E3 ubiquitin ligase, known to be involved in brassinosteroid signaling. This raises an important hypothesis that RGA1 may regulate tillering phenotypes by regulating brassinosteroid signaling pathways. The tillering phenotypes of altered TUD1 function under different conditions may reveal further insights in the light of our observations.

The *D10* gene, which we found to be down-regulated in the *rga1* mutant, codes for carotenoid-cleaving dioxygenase 8 (CCD8), a key enzyme involved in the biosynthesis of strigolactones. The loss of function of *D10* is known to result in increased tillering in rice^77^. Similarly, the *D14* gene (LOC_Os03g10620), which is also down-regulated in our *rga1* mutant, codes for an alpha/beta fold hydrolase family domain containing protein, and is reported to inhibit tiller development and yield by regulating strigolactone signaling^78^. Our finding that down-regulation of D10 and D14 in the *rga1* mutant is accompanied by increased tillering (Fig. 4), suggests the role of G-protein in strigolactone-mediated regulation of tillering. Similarly, the down-regulation of alpha amylase is known to change the grain morphology with decreased grain thickness and weight^79^. Our finding that alpha amylase is down-regulated in the *rga1* mutant (Fig. 4) brings out its role in alpha amylase-mediated grain development. This is also consistent with the literature on the role of G-protein signaling in regulating grain size in rice^80^. Over-expression of a homeobox domain containing protein (LOC_Os03g10210) has been shown to reduce the plant height and panicle development^81^. The up-regulation of this gene as we found in the *rga1* mutant (Fig. 4) may account for its reduced plant height and panicle development. Similarly, the up-regulation of AP2 domain containing protein (LOC_Os09g28440) in the *rga1* mutant may be associated with reduced plant height, as its expression remains low in the vegetative phase of normal wild type plant and negatively regulates plant height^82^. The phenotype of the *rga1* mutant may also be due to the altered regulation of hormone signaling pathways. The role of *RGA1* in brassinosteroid signaling is better studied as compared to other hormonal pathways^13,36^. We detected many DEGs associated with hormone biosynthesis as well as signaling and validated the regulation of two DEGs by RT-qPCR in the *rga1* mutant (Fig. 2), but the mechanisms of their regulation through *RGA1* need further elucidation**.**

We have shown that stress pathways constitute the second largest cluster of DEGs impacted by *RGA1*^28^ after metabolic regulation (Fig. 1). As an example of their role in stress phenotype, we showed here that the growth and biomass of *rga1* mutant were unimpacted by submergence under dark condition, while those of the WT were severely impacted (Fig. 6). It has been reported that *rga1* mutant displayed no significant cell death during submergence, indicating its role in submergence tolerance in rice^83^. The phenotype of the *rga1* mutant is similar to the SUB1 submergence tolerant genotype^84^, indicating that *RGA1* may function via SUB1 pathway during submergence. Pests and pathogens cause significant loss in global rice grain yield every year and the role of RGA1 in biotic stress response is known in principle^32^. However, this is the first time that a comprehensive listing of 83 *RGA1*-regulated genes involved in biotic stress response is being reported here in an important crop plant like rice. This enables further studies on the roles of *RGA1*-regulated DEGs in biotic stress responses, the notable among them being receptor like kinases (RLKs), phenyl ammonia lyase and PR genes.

Protein–protein interaction networks for G-protein signaling have been reported in Arabidopsis^50^, but this is the first time we report RGA1-regulated PPI networks and identified molecular complexes/sub-clusters. We also developed another PPI network using rice orthologs of the known interactors of G-protein signaling components in Arabidopsis. These networks revealed several interactors, including 43 validated in literature, and many possible effector molecules of G-protein signaling in rice.

In summary, our transcriptome profiling of the *rga1* mutant provides novel insights into the genome-wide role of G-protein (α subunit), including in many agronomically important traits in rice. They include tiller, heading date, panicle development, grain development and yield, N-response/NUE and biotic and abiotic stresses. Several differentially expressed genes that explain the observed phenotypic changes in the mutant have also been validated by RT-qPCR. N-responsive transcriptional regulatory network revealed many potential TFs and their target genes in the *rga1* mutant for further characterization of their role in N-response/NUE in rice. Predicted protein–protein interaction networks revealed several potential effectors and other intermediates of G-protein signaling in rice for the first time. Such improved understanding on the role of G-protein signaling in crop physiology and agronomic performance may aid in crop improvement in rice and possibly other crops.

## Methods

### Plant materials and growth condition

Wild type and d1 (Gα) mutant seeds of rice (*O. sativa* japonica Nipponbare) were procured from the Faculty of Agriculture, Kyushu University, Japan. Surface sterilization of the seeds was carried out using 70% ethanol and 0.01% Triton-X 100 and the seeds were grown on B-5 agar media in a controlled growth chamber maintained at 25 ± 2 °C, relative humidity of 70% and white light intensity of 1000 ± 100 lux with 12/12 h photoperiod. Leaves from 25 days old plants at the tertiary leaves stage were used for RNA isolation, microarray and qPCR validation.

For phenotypic trait analyses, wild type and mutant seedlings were transferred to separate pots containing either soil or 1:1 mixture of soilrite and vermiculite supplemented with 1X Arnon-Hoagland media to grow them for full life cycle under above conditions at a light intensity of 2800 ± 100 lux. Media was replenished after every 48 h. Various phenotypic traits such as tiller number, filled and unfilled seeds were measured in both wild type and mutant plants in at least 4 replicates and the leaf tissues were harvested during tillering, heading and grain filling stages for further analyses.

### RNA extraction, microarray analyses and data processing

RNA isolation, microarray analyses and data processing was done as described^28^. In brief, total RNA was extracted from frozen tissues and the quantity, quality, and suitability for microarray experiments were analyzed using Nanodrop spectrophotometer and Bioanalyzer (Agilent technologies, Santa Clara, USA). The Agilent 4 × 44 k rice array was used to perform the microarray experiments using total RNA isolated from two independent biological duplicates of both the wild type and d1 mutant. Low RNA Input Fluorescent Linear Amplification Kit (P/N: 5184-3523 Agilent, Santa Clara, CA) was used to transcribe the total RNA into Cyanine 3 (Cy3) labelled cRNA as per manufacturer’s instructions. RNeasy minikit (Qiagen) kit was used to purify the labeled cRNA and its specific activity was determined as a quality control for all the samples. Only samples that had specific activity > 8 were used for hybridization.

A reaction containing 1650 ng of each Cy3 labeled cRNA (41.8 µl), 10 × Blocking agent (11 µl) and 25 × Agilent fragmentation buffer (2.2 µl) was incubated at 60 °C for 30 min in the dark. The fragmented cRNA was mixed with 55 µl of 2 × hybridization buffer (Agilent) and hybridized for 17 h at 65 °C in an Agilent microarray hybridization chamber (SureHyb: G2534A) with hybridization oven at the Genotypic facility, Bangalore. The hybridized slides were washed using Agilent Gene expression Wash Buffer I for 1 min at room temperature, followed by a 1 min wash with Agilent Gene expression Wash Buffer II at 37 °C. Final washing was performed with acetonitrile and the air dried slides were scanned using Agilent scanner (G2565B) set at 100% laser power. Agilent Feature Extraction software (version 9.1) was used to extract the data, which were normalized as per the recommended “Per Chip and Per Gene Normalization” protocol. The data were analyzed using GeneSpring GX Version 11.5 (Genotypic Technology, Bangalore). Principal component analyses were used to obtain the correlation coefficients. Log_2_ fold change with geometric mean value of 1.0 and p-value < 0.05 were used as threshold values to define the differentially expressed genes (DEGs).

### Validation of selected *RGA1*-regulated genes by RT-qPCR

RT-qPCR analyses were carried out using total RNA to confirm the expression pattern of selected differentially regulated genes involved in different biological processes. To avoid the non-specific amplification from genomic DNA, we designed the primers spanning exon-exon junctions. About 5 µg of total RNAs extracted from the leaves of wild type and *rga1* mutants were reverse transcribed using R2D 1st strand cDNA synthesis kit (GCC Biotech, India) and PrimeScript 1st strand cDNA synthesis kit (TAKARA, Japan). The amplification step for RT-qPCR was performed using Aria Mx Real-time PCR System (Agilent technologies) in a 20 μl reaction containing 3 μl of diluted cDNA, 0.8 μl of forward and reverse gene specific primers (10 µM) and 10 μl of KAPA SYBR FAST Master Mix (2X) Universal (KapaBiosystems, USA). The relative accumulation of transcripts was calculated by the comparative C(T) method using actin (LOC_Os03g50885 and LOC_Os01g64630) gene as an internal control. Melting curve analyses of the amplicons was used to determine the specificity of qPCR reactions. The data were statistically analyzed using the GraphPad Prism 6 software. The experiments were performed using two biological and three technical triplicates.

### Functional classification of DEGs and MapMan pathways analyses

The DEGs were subjected to GO enrichment analyses using AgriGO with parameters [Analysis tool, Singular Enrichment Analysis (SEA); Species, *Oryza sativa* japonica; Suggested background, MSU7.0 gene ID (TIGR)]. The *p* value (< 0.05) and statistically stringent FDR value were used to detect the significantly enriched GO terms. The generated GO terms and associated values were taken to visualize the enrichments graphs using TreeMap (https://www.treemap.com/). The MapMan tool was used to highlight the changed pathways associated with DEGs that were assigned to different biological pathways (bins). The geomean fold change values (log_2_) of DEGs were used to represent the coloured boxes in the map.

### Subcellular localization of DEGs

The amino acids sequences of the DEGs were retrieved from RGAP database and subjected to CELLO program (http://cello.life.nctu.edu.tw/) to predict the subcellular distribution using default parameters for eukaryotes. The highest reliability score obtained from CELLO prediction was used to annotate the localization of a protein.

### Construction of protein–protein interaction networks and detection of molecular complexes

The list of interacting proteins for the DEGs analyzed in this study were retrieved from STRING (https://string-db.org/), MCDRP (http://www.genomeindia.org/biocuration/), BioGRID (https://thebiogrid.org/) and PRIN (http://bis.zju.edu.cn/prin/) databases. In this study, we considered only those interactions demonstrated by experiments and mapped the DEGs to the protein–protein interaction (PPI) networks. Based on the experimental score, we constructed PPI network using Cytoscape version 8.0. To detect the molecular complexes, we used molecular complex detection (MCODE) plugin in the Cytoscape network.

### Submergence stress evaluation

Intact seeds of wild type and *rga1* mutants were germinated for 5 days on muslin cloth presoaked with ultrapure water. Seedlings of similar growth were submerged in glass tubes containing ultrapure water and kept at 25 °C for 6 more days in the dark along with non-submerged controls and used for phenotypic measurements.

### Toluidine blue permeability test

Toluidine blue (TBO) staining is known to examine the cuticular properties of plant^85^. Experiments were performed as described earlier^86,87^ using seeds^88^. TBO solution was prepared with 10 mM sodium citrate, pH 4.4; 0.05w/v toluidine blue and 0.4 v/v tween 20. Ten seeds each of wild type and *rga1* were immersed in the solution in microcentrifuge tubes and incubated at room temperature for not more than 3 min. They were gently rinsed with water with a pipette for 3 min to remove unbound stain. They were treated with 80% ethanol and incubated at 37 ºC for 2 h and absorbance readings were taken at 626 and 430 nm using Agilent Cary 60 spectrophotometer.

## Supplementary Information

Supplementary Information 1.

Supplementary Information 2.
