# Supplementary material for: Heterotrimeric G-protein α subunit (RGA1) regulates tiller development, yield, cell wall, nitrogen response and biotic stress in rice
Source: Sci Rep. 2021 Jan 27;11:2323. doi: 10.1038/s41598-021-81824-1 (PMC7840666; doi:10.1038/s41598-021-81824-1)
Supplement: Supplementary file 1 — Supplementary Information 1. [file 41598_2021_81824_MOESM1_ESM.pdf]

Heterotrimeric G-protein α subunit (*RGA1*) regulates tiller development, yield, cell wall, nitrogen response and biotic stress in rice

Ravi Ramesh Pathak, Vikas Kumar Mandal, Annie Prasanna Jangam, Narendra Sharma, Bhumika Madan, Dinesh Kumar Jaiswal, Nandula Raghuram

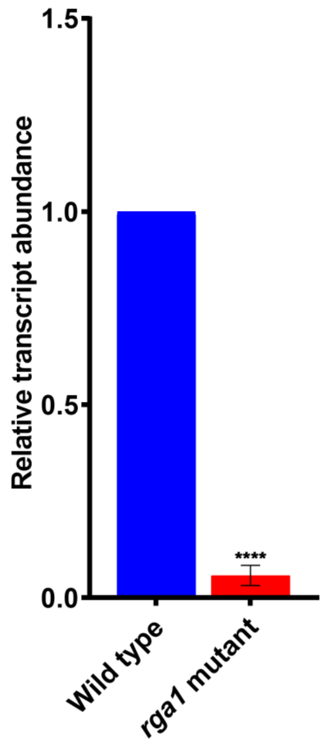

Figure S1: Relative expression of *RGA1*(LOC_Os05g26890) transcript in the wild type and *rga1* mutant. Total RNA isolated from the leaves of 52 days old wild type and *rga1* mutant plants grown under standard condition of temperature, humidity and light intensity (7000±100 Lux). Actin (LOC_Os01g64630) was used as an internal control gene for data normalization. The experiment was performed using two biological and three technical replicates. Relative quantification of transcript is represented with Mean±SE. The unpaired t-test analysis was performed using the GraphPad Prism 6.0 (https://www.graphpad.com/scientific-software/prism/). ****P<0.0001.

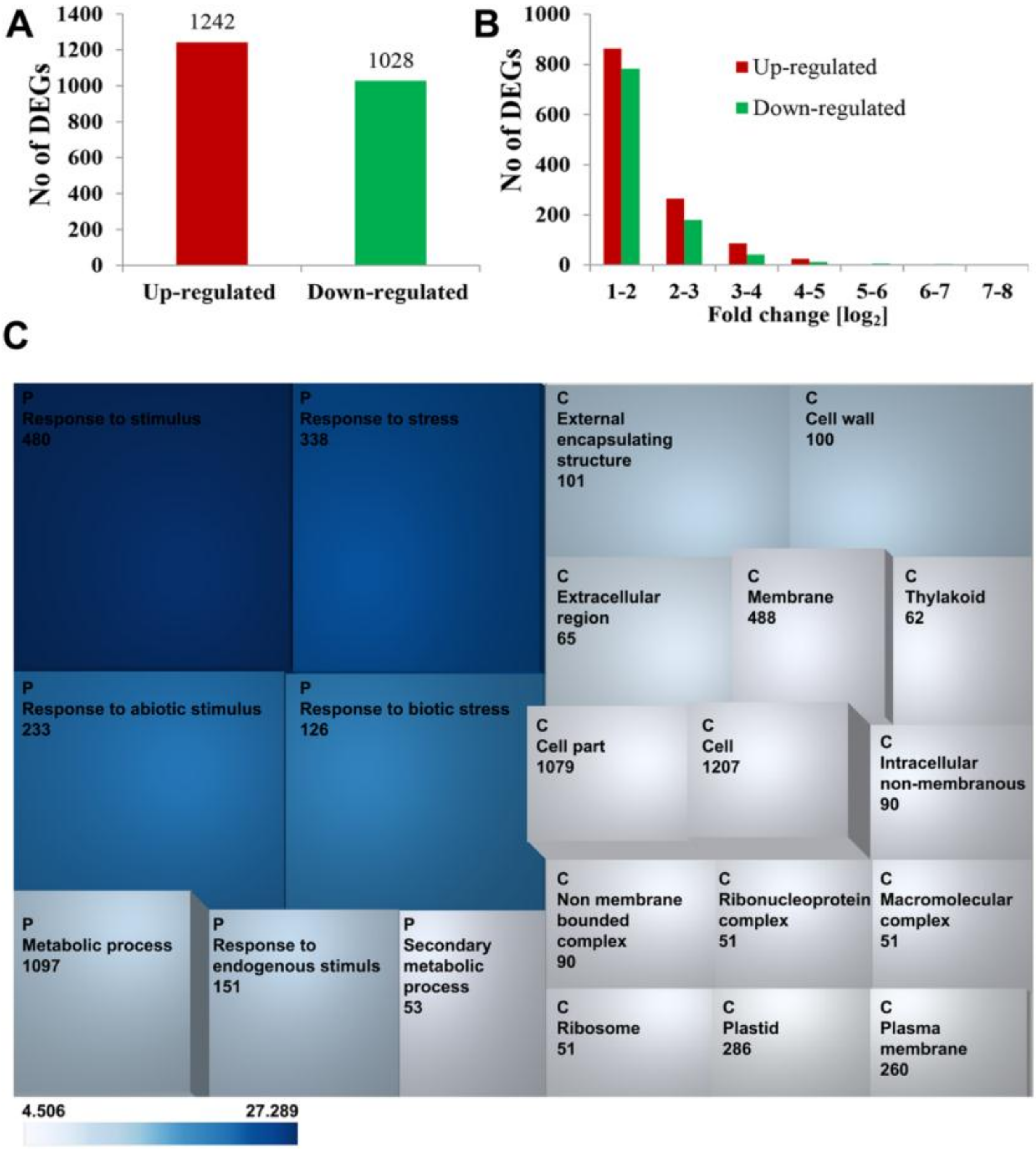

Figure S2. Distribution of *RGA1*-regulated DEGs and their Gene Ontology-based functional annotation. (A) Total number of up- and down-regulated DEGs identified in the *rga1* mutant. (B) The distribution of DEGs according to their fold change value. (C) Functional annotation of the DEGs using AgriGO2.0 tool (http://systemsbiology.cau.edu.cn/agriGOv2/). The p-value, FDR and the numbers of DEGs assigned to each functional annotation were retrieved from AgriGO analyses and visualized using TreeMap (https://www.treemap.com/). The grouping was performed using ontology; size is proportional to p-value ($-\log_2$), and coloured by FDR value ($-\log_2$).

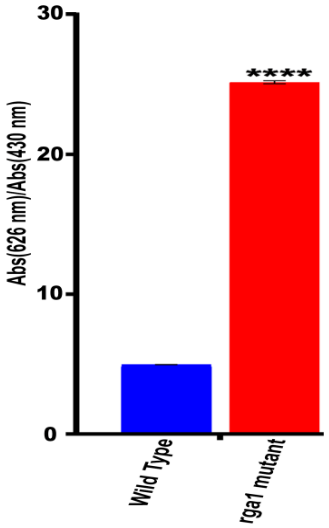

Figure S3. Toluidine blue permeability analysis. Spectrophotometric analysis for lignin concentration by toluidine blue staining indicated enhanced permeability of cuticle in 10 seeds of *rga1* mutant as compared to wild type. Unpaired t-test was performed to check the significance of difference between the mean values of individual groups (****p value < 0.0001).

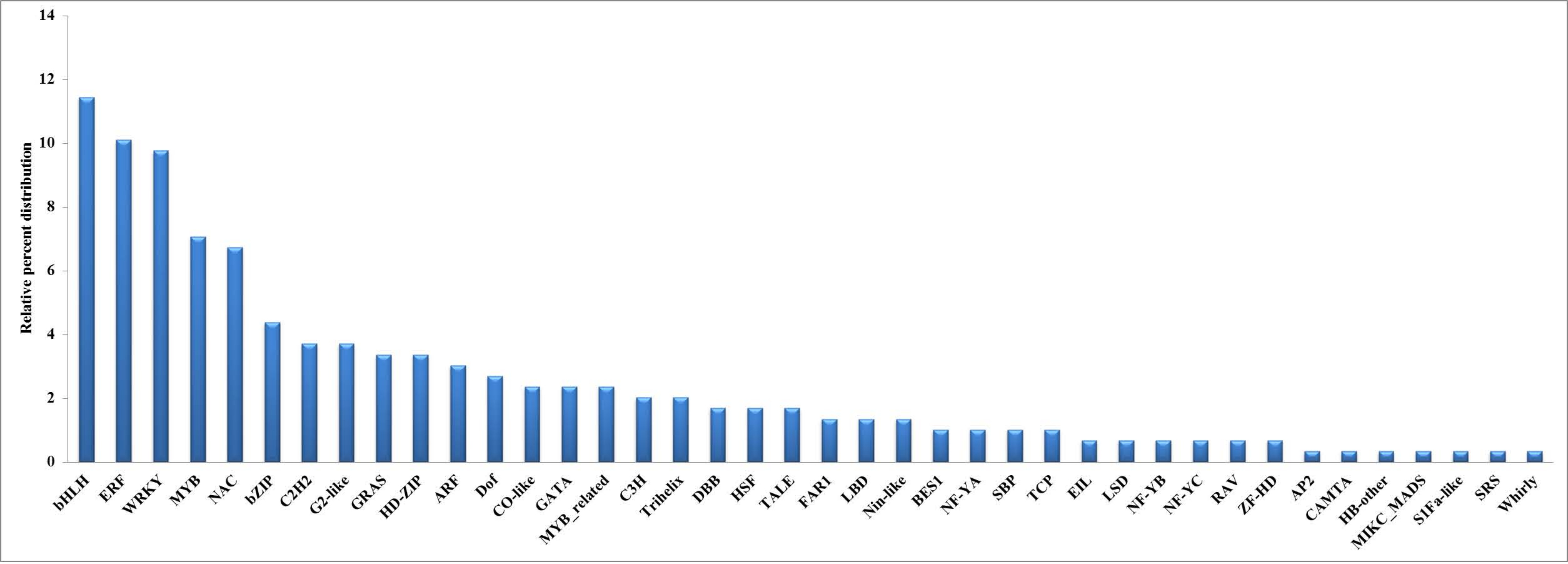

Figure S4. Relative distribution of transcription factors differentially regulated in rice rga1 mutant. Transcription factors were mined by searching the DEGs in RGAP database and classified accordingly.

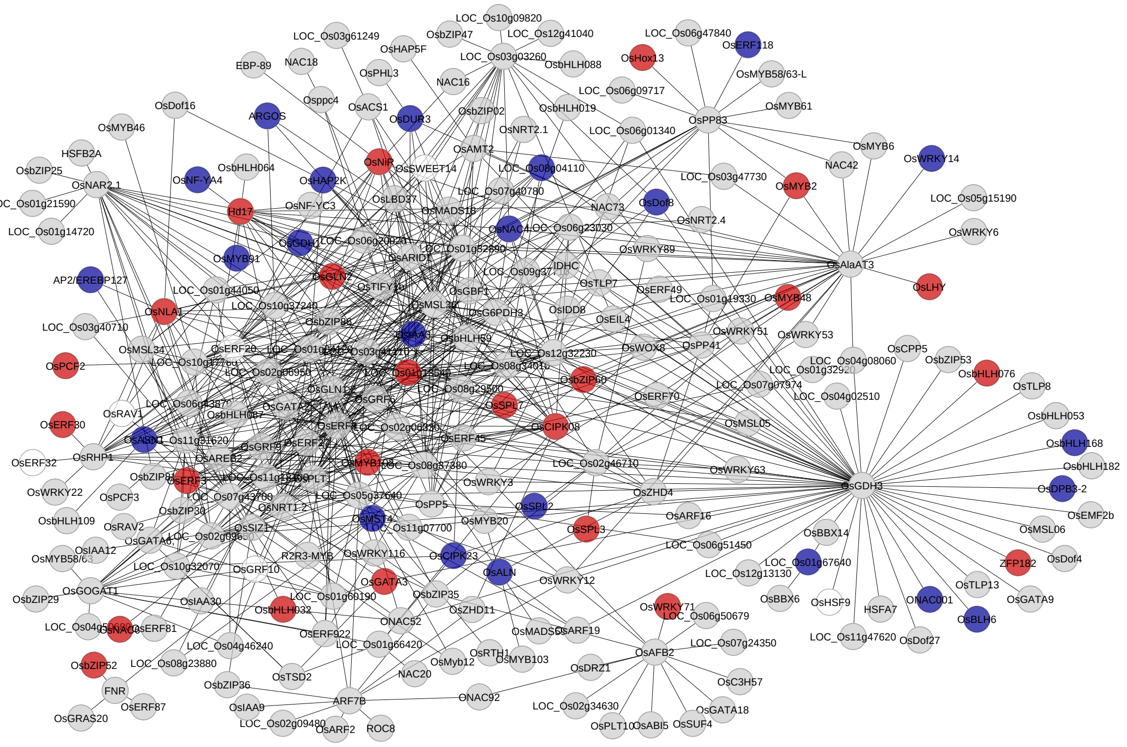

Figure S5. Nitrate-regulated transcriptional regulatory network in the rga1 mutant. Transcriptional regulatory network was generated using nitrate-regulated transcriptional regulatory network known in Arabidopsis (Gaudinier et al., 2018, Nature). TRN was constructed in Cytoscape version 8.0.0 (https://cytoscape.org/) and expressions profile of DEGs were mapped onto the networks. The red and blue nodes represent the up- and down-regulated DEGs and grey colour nodes are not DEGs

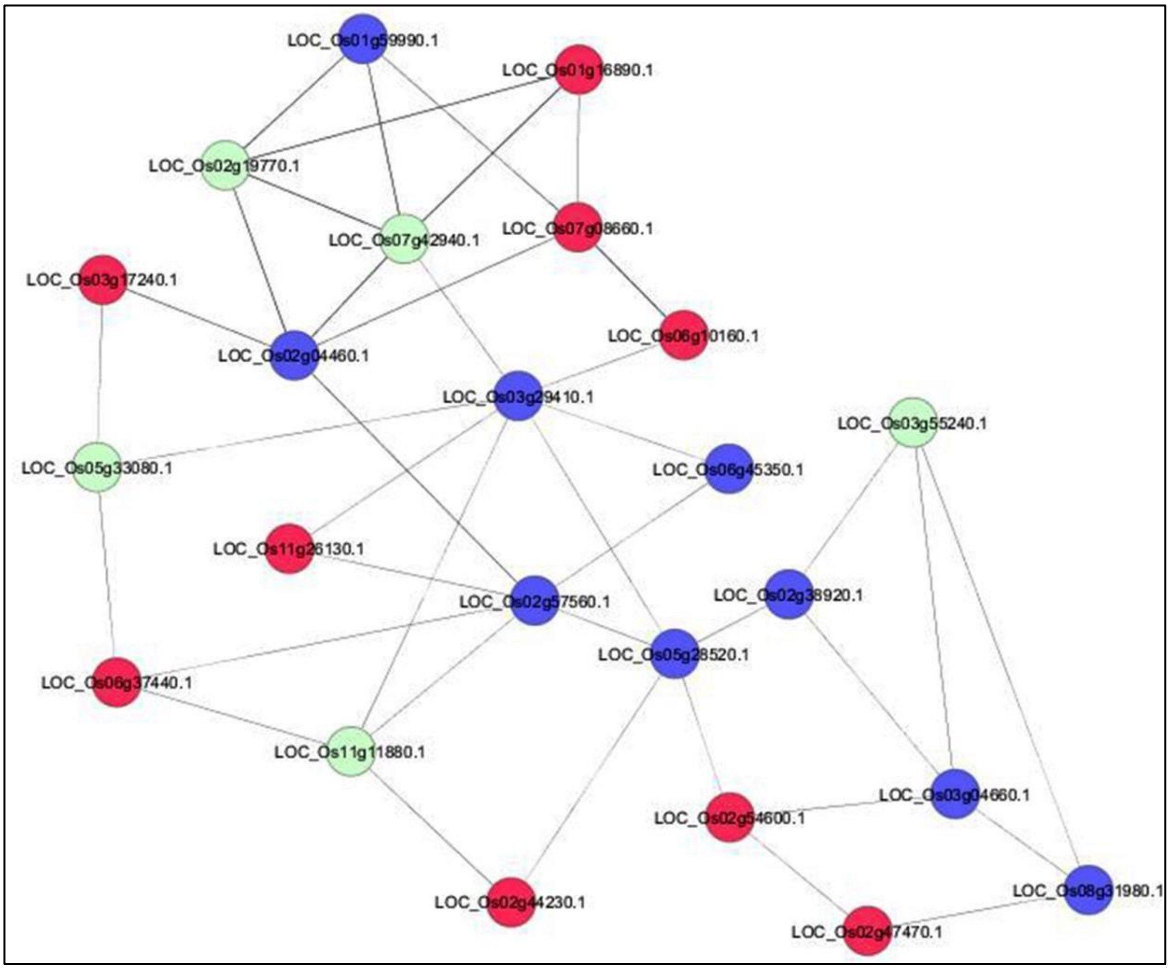

Figure S6. RGA1-regulated subcluster-3/molecular complex-3 associated with different cellular pathways. All the DEGs identified in our study were used to retrieve the experimentally validated interactors from BioGRID, STRING and other databases. The protein-protein interaction (PPI) networks were constructed using DEGs associated interactors in Cytoscape and molecular complexes were identified using the MCODE plugin in Cytoscape. The red and blue nodes represent the up- and down-regulated DEGs, respectively. Interactors that are not DEGs are assigned with light colour.

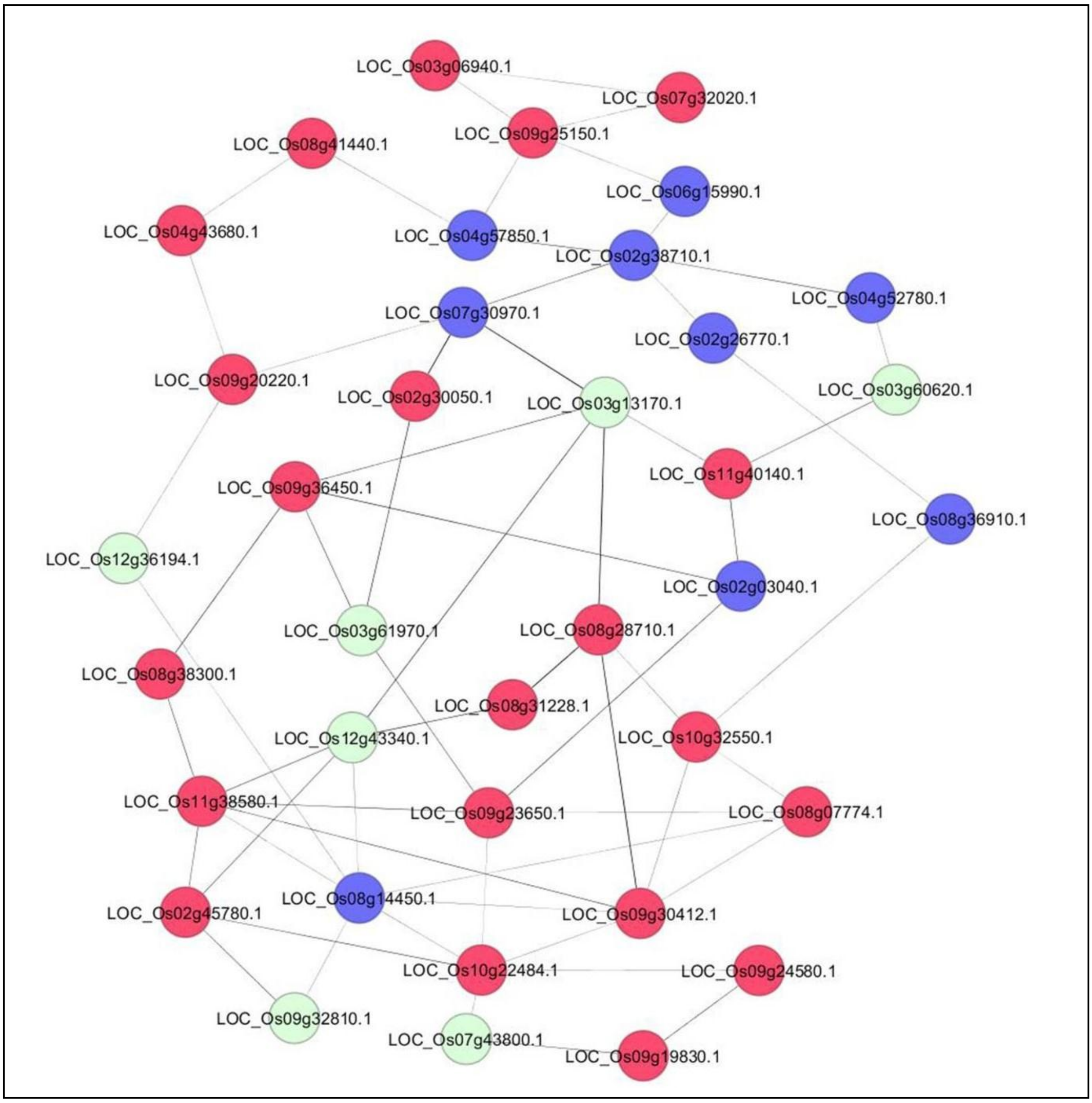

Figure S7. RGA1-regulated subcluster-4/molecular complex-4 associated with different cellular pathways. All the DEGs identified in our study were used to retrieve the experimentally validated interactors from BioGRID, STRING and other databases. The protein-protein interaction (PPI) networks were constructed using DEGs associated interactors in Cytoscape and molecular complexes were identified using the MCODE plugin in Cytoscape. The red and blue nodes represent the up- and down-regulated DEGs, respectively. Interactors that are not DEGs are assigned with light colour.

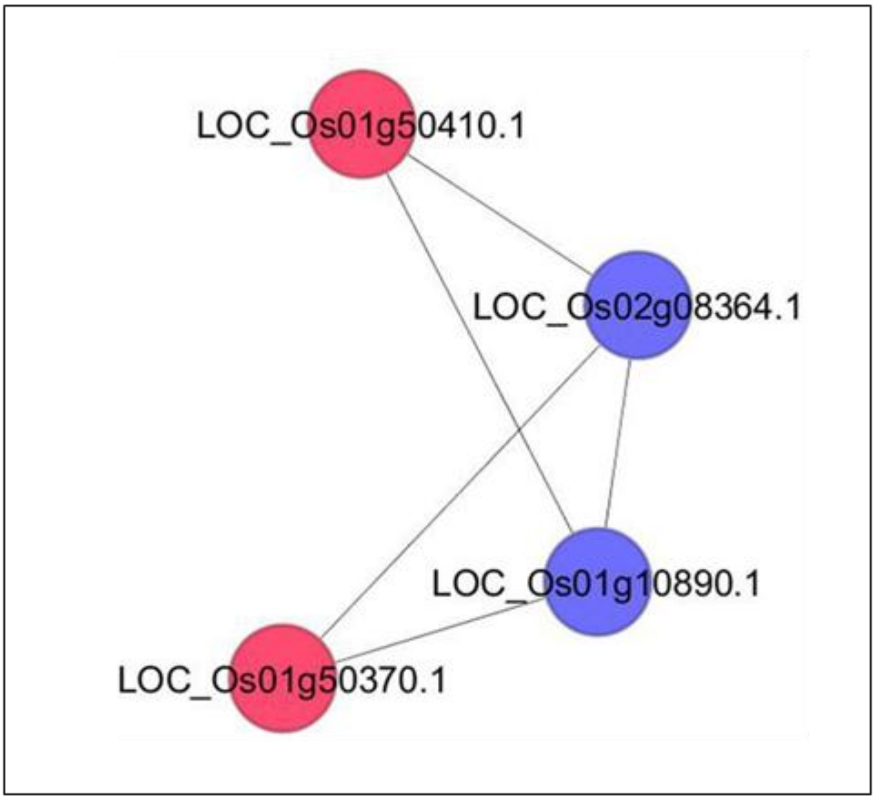

Figure S8. RGA1-regulated subcluster-5/molecular complex-5 associated with different cellular pathways. All the DEGs identified in our study were used to retrieve the experimentally validated interactors from BioGRID, STRING and other databases. The protein-protein interaction (PPI) networks were constructed using DEGs associated interactors in Cytoscape and molecular complexes were identified using the MCODE plugin in Cytoscape. The red and blue nodes represent the up- and down-regulated DEGs, respectively. Interactors that are not DEGs are assigned with light colour.

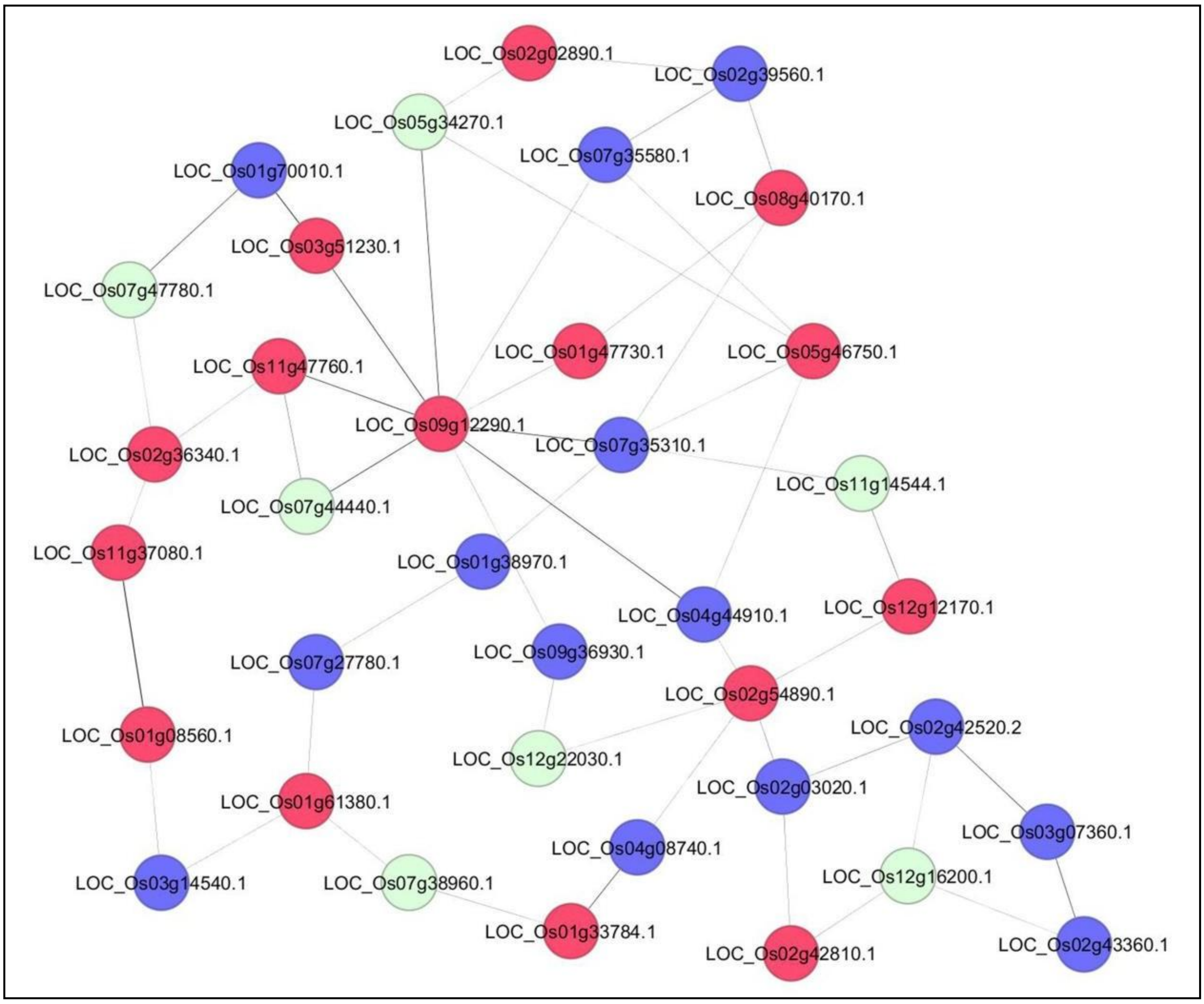

Figure S9. RGA1-regulated subcluster-6/molecular complex-6 associated with different cellular pathways. All the DEGs identified in our study were used to retrieve the experimentally validated interactors from BioGRID, STRING and other databases. The protein-protein interaction (PPI) networks were constructed using DEGs associated interactors in Cytoscape and molecular complexes were identified using the MCODE plugin in Cytoscape. The red and blue nodes represent the up- and down-regulated DEGs, respectively. Interactors that are not DEGs are assigned with light colour.

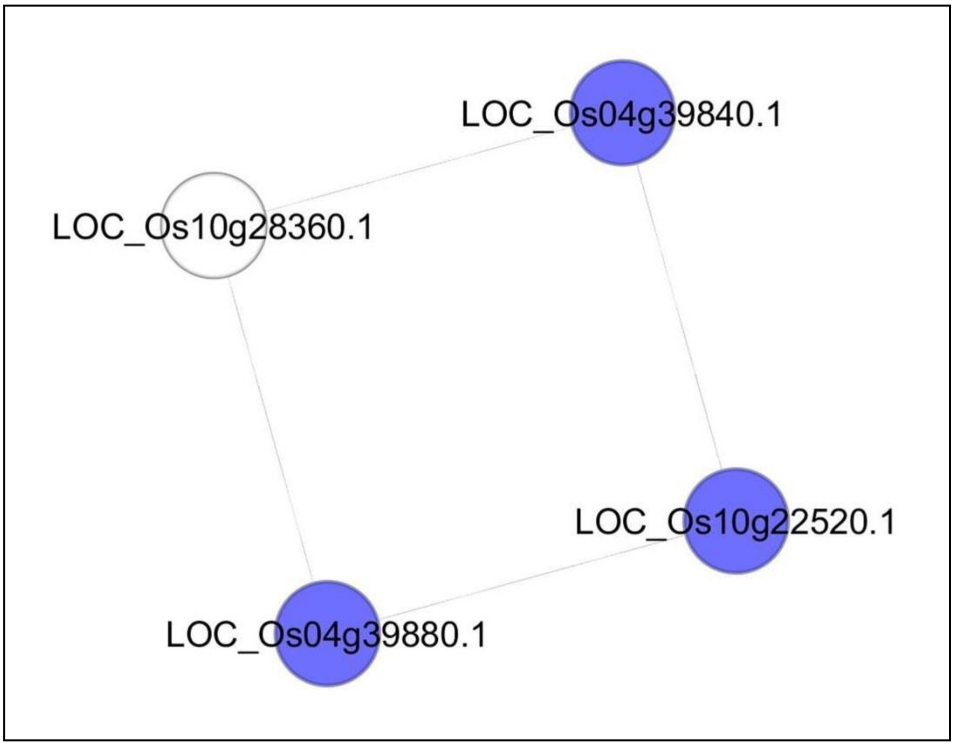

Figure S10. RGA1-regulated subcluster-7/molecular complex-7 associated with different cellular pathways. All the DEGs identified in our study were used to retrieve the experimentally validated interactors from BioGRID, STRING and other databases. The protein-protein interaction (PPI) networks were constructed using DEGs associated interactors in Cytoscape and molecular complexes were identified using the MCODE plugin in Cytoscape. The red and blue nodes represent the up- and down-regulated DEGs, respectively. Interactors that are not DEGs are assigned with light colour.

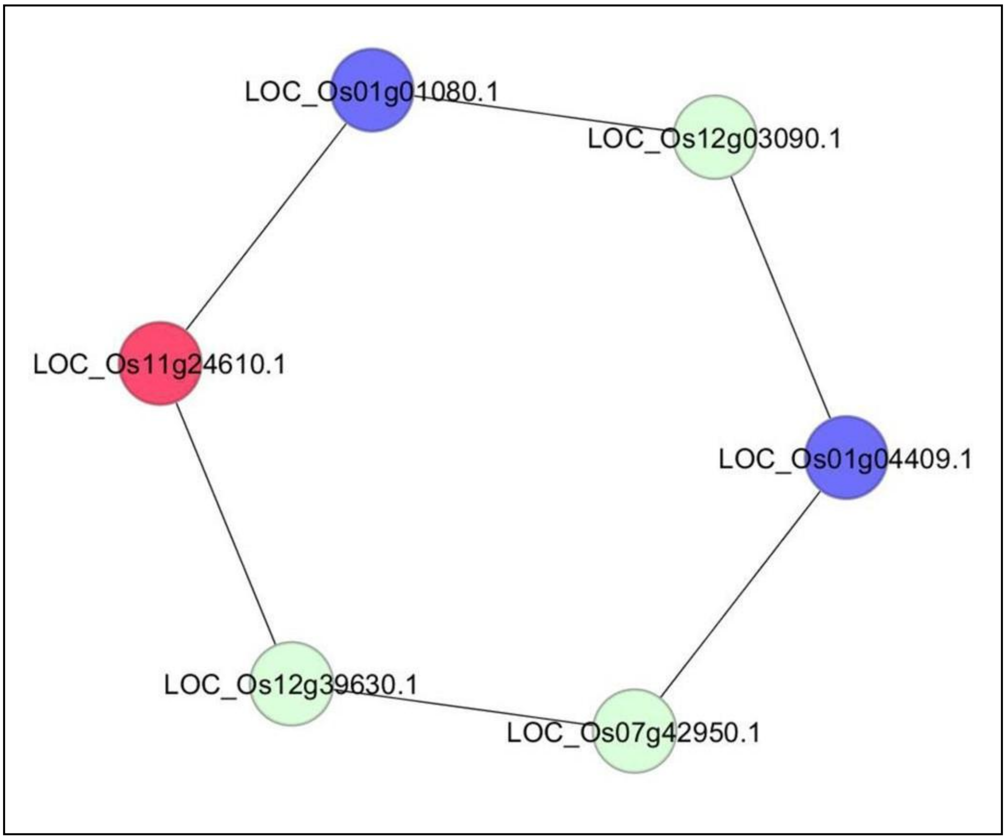

Figure S11. RGA1-regulated subcluster-8/molecular complex-8 associated with different cellular pathways. All the DEGs identified in our study were used to retrieve the experimentally validated interactors from BioGRID, STRING and other databases. The protein-protein interaction (PPI) networks were constructed using DEGs associated interactors in Cytoscape and molecular complexes were identified using the MCODE plugin in Cytoscape. The red and blue nodes represent the up- and down-regulated DEGs, respectively. Interactors that are not DEGs are assigned with light colour.

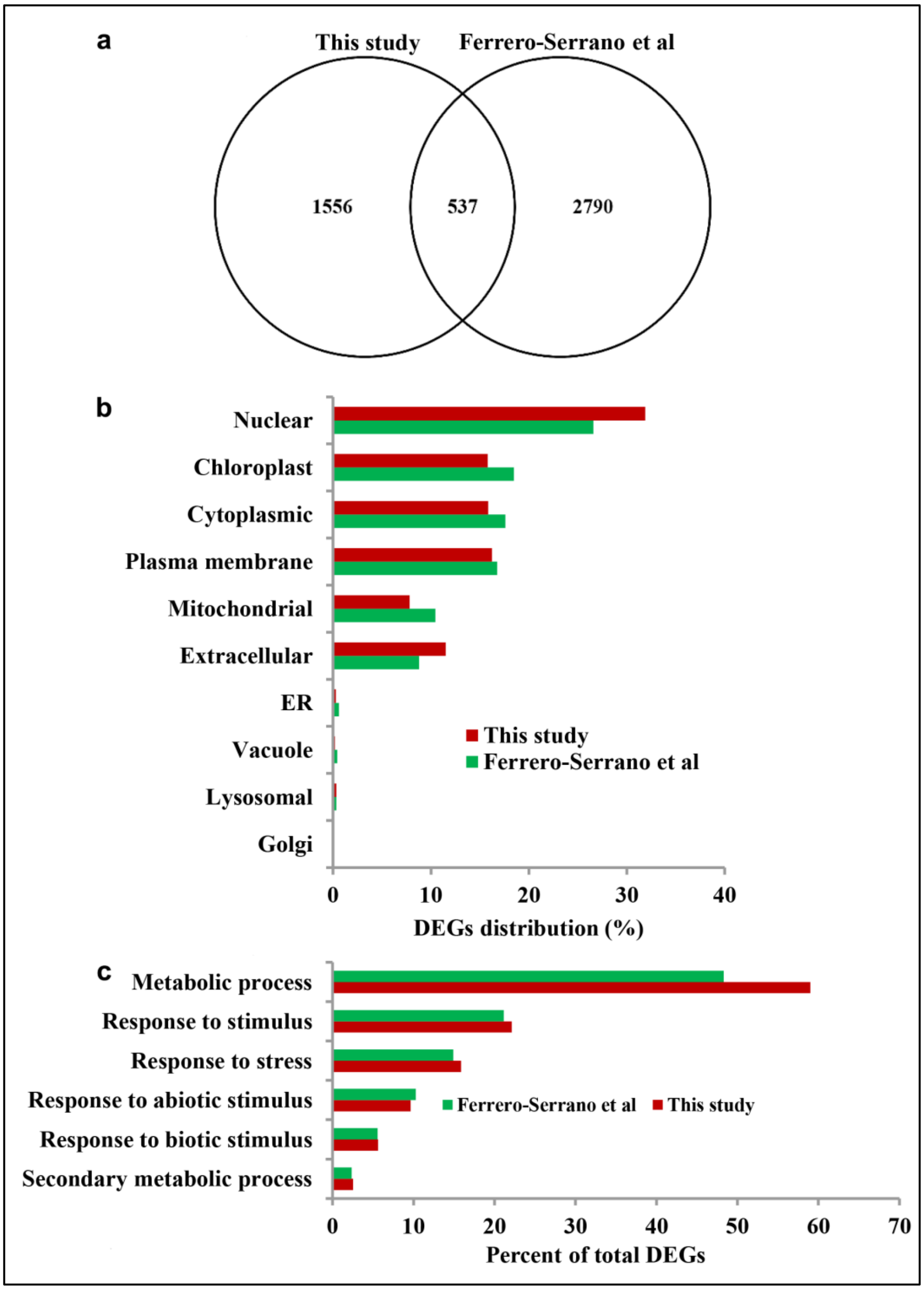

Figure S12. Parallel comparison of *RGA1*-regulated transcriptomic datasets identified in our study and Ferrero-Serrano et al., (2018). (a) Venn diagram showing the common and exclusive DEGs identified in our study and Ferrero-Serrano et al., (2018). (b) Bar diagram showing the subcellular localization of DEGs indentified in both the studies using CELLO program (http://cello.life.nctu.edu.tw/). (C) Comparison of GO biological processes identified in both the studies using AgriGO2.0 tool.

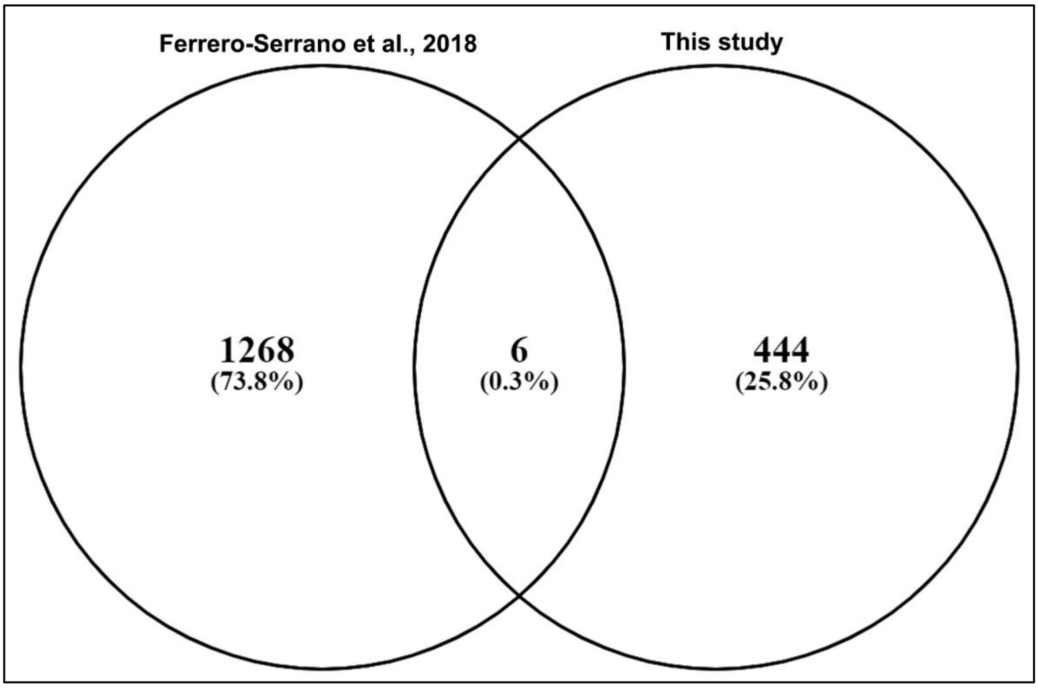

Figure S13. Venn selection represent the interactors associated with exclusive DEGs identified in this study and other (Ferrero-Serrano et al., 2018).
